# Mining the Vavilov wheat diversity panel for new sources of adult plant resistance to stripe rust

**DOI:** 10.1007/s00122-022-04037-8

**Published:** 2022-02-03

**Authors:** Dilani T. Jambuthenne, Adnan Riaz, Naveenkumar Athiyannan, Samir Alahmad, Wei Ling Ng, Laura Ziems, Olga Afanasenko, Sambasivam K. Periyannan, Elizabeth Aitken, Greg Platz, Ian Godwin, Kai P. Voss-Fels, Eric Dinglasan, Lee T. Hickey

**Affiliations:** 1 Queensland Alliance for Agriculture and Food Innovation, The University of Queensland, St Lucia, QLD Australia; 2 Commonwealth Scientific and Industrial Research Organization (CSIRO), Agriculture and Food,, Canberra, ACT Australia; 3 Department of Plant Resistance To Diseases, All Russian Research Institute for Plant Protection, St Petersburg, Russia 196608; 4 School of Agriculture and Food Sciences, The University of Queensland, St Lucia, QLD Australia; 5 Department of Agriculture and Fisheries, Hermitage Research Facility, Warwick, QLD Australia

## Abstract

**Key message:**

Multi-year evaluation of the Vavilov wheat diversity panel identified new sources of adult plant resistance to stripe rust. Genome-wide association studies revealed the key genomic regions influencing resistance, including seven novel loci.

**Abstract:**

Wheat stripe rust (YR) caused by *Puccinia striiformis* f. sp*. tritici* (*Pst*) poses a significant threat to global food security. Resistance genes commonly found in many wheat varieties have been rendered ineffective due to the rapid evolution of the pathogen. To identify novel sources of adult plant resistance (APR), 292 accessions from the N.I. Vavilov Institute of Plant Genetic Resources, Saint Petersburg, Russia, were screened for known APR genes (i.e. *Yr18*, *Yr29*, *Yr46*, *Yr33*, *Yr39* and *Yr59*) using linked polymerase chain reaction (PCR) molecular markers. Accessions were evaluated against *Pst* (pathotype 134 E16 A + Yr17 + Yr27) at seedling and adult plant stages across multiple years (2014, 2015 and 2016) in Australia. Phenotypic analyses identified 132 lines that potentially carry novel sources of APR to YR. Genome-wide association studies (GWAS) identified 68 significant marker–trait associations (*P* < 0.001) for YR resistance, representing 47 independent quantitative trait loci (QTL) regions. Fourteen genomic regions overlapped with previously reported *Yr* genes, including *Yr29*, *Yr56*, *Yr5*, *Yr43*, *Yr57*, *Yr30*, *Yr46, Yr47*, *Yr35*, *Yr36*, *Yrxy1*, *Yr59*, *Yr52* and *YrYL*. In total, seven QTL (positioned on chromosomes 1D, 2A, 3A, 3D, 5D, 7B and 7D) did not collocate with previously reported genes or QTL, indicating the presence of promising novel resistance factors. Overall, the Vavilov diversity panel provides a rich source of new alleles which could be used to broaden the genetic bases of YR resistance in modern wheat varieties.

**Supplementary Information:**

The online version contains supplementary material available at 10.1007/s00122-022-04037-8.

## Introduction

Wheat is one of the most widely cultivated cereal crops worldwide, with an annual production of approximately 761 million tonnes (Crop Prospects and Food situation 2020). Global wheat yields are threatened by climate change (Asseng et al. 2015; Barlow et al. 2015) and rapidly evolving pathogens causing serious outbreaks of diseases, including rusts (Chaves et al. 2013). Among the rusts, stripe rust (YR) caused by *Puccinia striiformis* f.sp. *tritici* (*Pst*) is an economically important disease that has caused several major epidemics worldwide, resulting in significant production losses (Sanin and Nazarova 2010; Hovmoller et al. 2011; Ellis et al. 2014b; Xia et al. 2016a; Rahmatov 2016; Ali et al. 2017). Historically, YR was prevalent in cooler climates; however, the majority of wheat-growing areas in the world have now become prone to YR (Ali et al. 2014; Hubbard et al. 2015). Currently, 88% of global wheat production is under threat to YR, which accounts for annual losses of more than one billion US dollars (Beddow et al. 2015). Deployment of YR-resistant varieties is the preferred method of rust disease management because it is cost-effective and reduces the reliance on fungicides (Chen 2005a).

Genetic resistance to YR is broadly categorized into two major classes: All stage resistance (ASR) (e.g. R-genes) and adult plant resistance (APR-genes). ASR is often underpinned by a single gene with a large effect that provides effective resistance at all stages of plant growth. The R-gene interacts with the pathogen in a gene-for-gene relationship (Flor 1971) and therefore is commonly referred to as “race-specific” resistance (Ellis et al. 2014a). When deployed in a variety grown on a large scale, strong selection pressure is exerted on the pathogen population to select for mutations that overcome the resistance mechanism. Therefore, this type of resistance is often rendered ineffective within just 3–5 years. In contrast, APR is typically controlled by multiple genes, each with minor or partial effect, and is usually best expressed at adult growth stages. Most of the well-characterised APR genes are race nonspecific and usually confer a “slow rusting” phenotype, which is known to be more durable (Lagudah 2011a; Ellis et al. 2014a; Mundt 2014). Importantly, APR genes can contribute to high levels of resistance through additive or epistatic effects (Sorensen et al. 2014). Some of the genes, such as pleiotropic APRs and high temperature adult plant (HTAP) resistance genes, are highly valuable in breeding programmes. For instance, APR genes *Yr18*, *Yr29*, *Yr30* and *Yr46* confer pleiotropic resistance to YR, leaf rust, stem rust and powdery mildew of wheat (Lan et al. 2015), while *Yr18*, *Yr29*, *Yr36*, *Yr39* and *Yr52* exhibit HTAP resistance (Chen 2013b).

To date, 83 *Yr* resistance genes (*Yr1*–*83*) have been officially designated, along with 47 genes that have been temporarily named (McIntosh et al. 2019). According to the available information, most are classified as R-genes, whereas only 18 are classified as APR genes (Wu et al. 2016). Notably, only three APR genes *Yr18*/*Lr34* (Krattinger et al. 2009), *Yr36* (Fu et al. 2009), and *Yr46* (Moore et al. 2015a) have been cloned to date (Liu et al. 2015). The majority of the R-genes, which have been deployed in various wheat varieties, are no longer effective due to the emergence of virulent pathotypes (Hovmoller et al. 2011). For instance, a large number of predominant races evolved from the year 2000 onwards and displayed added virulence to numerous resistance genes such as *Yr2*, *Yr6*, *Yr7*, *Yr8*, *Yr9*, *Yr10*, *Yr17*, *Yr27*, *Yr43* and *Yr44* (Wan and Chen 2014). Virulence was also reported for some of the APR genes (Sorensen et al. 2014). Therefore, an additional level of durable genetic resistance could be achieved by pyramiding both seedling and APR genes in future varieties (Mundt 2014). Hence, the discovery of new sources of genetic resistance is a priority for wheat research, and the successful integration of new technologies in crop improvement programmes is important to achieve long-term rust control.

Plant genetic resources that are stored in gene banks worldwide are a valuable source of genetic diversity for biotic and abiotic stresses (Rao 2004b). Among these, wheat landraces and wild relatives are valuable sources of novel alleles for YR resistance (Sthapit et al. 2014, 2017; Manickavelu et al. 2016; Pasam et al. 2017). In previous studies, three important *Yr* genes (*Yr47*, *Yr51* and *Yr57*) have been successfully characterized from wheat landraces in the Watkins collection (Bansal et al. 2011; Randhawa et al. 2015). Another historically important germplasm collection is preserved at the N.I. Vavilov Institute of plant genetic resources (VIR) in Saint Petersburg, Russia, which holds ~ 38,430 wheat accessions. The collection comprises 76% bread wheat, 16% durum wheat and 7.9% wild and primitive wheats from diverse geographical origins (i.e. Africa, east and west Asia, the USA, Canada, Central and South America, Europe) and of diverse biological status, including wild forms, local cultivars, breeding lines, mutants and artificial allopolyploids (Mitrofanova 2012). While the VIR collection is yet to be explored for YR resistance, previous studies have highlighted the genetic variation for a number of biotic stresses (Mitrofanova 2012; Sadovaya et al. 2015; Riaz et al. 2016a, 2018).

To unravel the genetic architecture of rust resistance, bi-parental linkage mapping studies are traditionally performed (Yang et al. 2017). However, high costs associated with population development, poor mapping resolution due to low recombination and the constraint of low allelic diversity are some of the limitations associated with the linkage mapping approach (Flint-Garcia, 2013). Alternatively, a GWAS (Genome-wide association study) approach can be applied to a collection of accessions or a natural population. It offers broader allele coverage and higher mapping resolution due to historical recombination events among the panel of lines (Brachi et al., 2011a). This helps to localise the association signals to smaller regions within the chromosome and supports more efficient identification of candidate gene(s). However, applying GWAS to germplasm collections can be challenging because of population structure, which can result in spurious correlations between markers and traits (Gupta 2014; Kulwal et al. 2019; Yang et al. 2017). Another drawback is the low detection power of rare alleles with larger effects or multi-allelic variants with minor effects. Thus, an ideal GWAS analysis requires a large population size, high marker density and a mixed linear model to detect true genotype–phenotype associations (Bulli et al. 2016a). GWAS was initially successfully implemented for rust resistance in hexaploid wheat by Crossa et al. (2007b). Since then, it has been widely used in several studies to detect genomic regions associated with YR resistance in spring, winter, synthetic wheat germplasm, and landraces (Zegeye et al. 2014; Jighly et al. 2015; Maccaferri et al. 2015b; Naruoka et al. 2015; Bulli et al. 2016a; Godoy et al. 2017; Liu et al. 2017).

In this study, we evaluated 292 hexaploid bread wheat accessions from the VIR against *Pst* to identify novel sources of YR resistance. We applied GWAS to identify key genomic regions that could support the development of new cultivars incorporating durable resistance to YR.

## Materials and methods

### Plant material

This study examined the Vavilov wheat diversity panel for resistance to YR. The composition of the panel was previously described by Riaz et al. (2017). Briefly, it includes 292 bread wheat accessions, including 136 landraces, 32 cultivars, 10 breeding lines and 115 lines with unknown cultivation classification, which were collected over a 70-year period (Online Resource 1). A total of 206 lines have origin information, whereas the origin is unknown for the remaining 89 lines. Notably, the diversity panel is both morphologically and genetically more diverse compared to Australian and CIMMYT elite wheat materials (Riaz et al. 2017).

### Pathogen

Pathotype 134 E16 A + was used to screen the diversity panel because it was the most virulent and widespread pathotype in Australia during the experimental period. The pathotype 134 E16 A + was first detected in Western Australia in 2002 where it initially displayed virulence for *Yr6*, *Yr7*, *Yr8*, *Yr9*, and *YrA* (Wellings et al. 2003) and gradually acquired virulence for *Yr10* and *Yr17* (Wellings 2007). Virulence for *Yr27* was first reported in 2008, and combined virulence for *Yr17* and *Yr27* in pathotype E16A + Yr17 + Yr27 + was detected in 2010 (Randhawa et al. 2015).

### PCR marker screening for known APR genes

A subset of 283 wheat accessions were screened for known APR genes. The accessions were screened by a PCR reaction, using markers corresponding to *Yr18*, *Yr29*, *Yr46*, *Yr33*, *Yr39* and *Yr59*. Marker screening for *Yr18*, *Yr29* and *Yr46* (Riaz et al. 2016b) was performed with gene-specific cleaved amplified polymorphic sequence (CAPS) markers cssfr5 (Lagudah et al. 2009), csLV46 (unpublished) and a gene-specific single-nucleotide polymorphic marker SNP1-TM4 (Moore et al. 2015b), respectively. The genes *Yr33* and *Yr59* were evaluated, each with two closely linked markers to detect their presence or absence. SSR markers gwm111 and gwm437 were used to identify *Yr33* (Zahravi et al. 2003), whereas the SSR marker barc32 and resistance gene analog polymorphism (RGAP) marker wmc557 were used to identify *Yr59* (Zhou et al. 2014). The *Yr39* gene was evaluated using the RGAP marker wgp45 (Lin and Chen 2007). PCR was carried out in a 20 µl reaction containing upto 100 ng of genomic DNA, 1X GoTaq Flexi buffer, 1.5 mM MgCl_2_, 200 µM dNTP, 200 nM of both forward and reverse primers and 1U of *Taq* polymerase (M829B, Promega, the USA). A touch down PCR cycling condition was used as follows: denaturation at 94 °C for 30 s; annealing at 62 °C for 30 s, decreasing by 0.5 °C per cycle; extension at 72 °C for 1 min followed by repeating these steps for 10 cycles; after enrichment, the programme continued for 29 cycles as follows: 94 °C for 30 s, 58 °C for 30 s and 72 °C for 40 s. Amplified products were evaluated using agarose gel electrophoresis.

### Phenotyping for seedling resistance

All accessions were evaluated for *Pst* resistance at the seedling stage in a glasshouse (GH) at the University of Queensland, St Lucia, Queensland, Australia. The experiment was conducted using a complete randomised design with three replicates. Three to four seeds per accession were sown at four different positions around the circumference of 140 mm ANOVApots® filled with potting media. Seedlings were raised under glasshouse conditions maintained at day/night temperature of 22/17 °C using a natural 12 h diurnal photoperiod. Eleven-day-old seedlings (i.e. two-leaf stage) were inoculated with *Pst* pathotype 134E16 A + Yr17 + Yr27 according to the air-brush protocol reported by Hickey et al. (2012). Seedlings were evaluated after 14 days post-inoculation based on 0–4 Stakman et al. (1962) scale which contains both numbers (e.g. 0, 1…4) and symbols (e.g. +). The numbers represent the disease score: highly resistant (HR, 0–1), moderately resistant (MR, 2), moderately susceptible (MS, 3), and susceptible (S, 4).

### Phenotyping for adult plant resistance

A total of 292 accessions of the Vavilov wheat diversity panel were evaluated across three years (2014, 2015 and 2016) at The University of Queensland Gatton Research Station, Queensland, Australia. Wheat lines were sown in the field as un-replicated hill plots. The hill plots were 0.5 m apart along the direction of sowing and in two rows positioned between two spreader rows of the very susceptible genotype, Morocco (Online Resource 2a). To establish YR in the nursery, wheat seedlings that were raised in the glasshouse, infected with *Pst*, and were transplanted among the spreader rows approximately one month after sowing the nursery. The reaction to YR was evaluated using a modified Cobb’s scale (Peterson et al., 1948) and grouped on a 0‒9 scale in 2014, a 1‒9 scale (Bariana et al., 2007) was used in 2015 and 2016, the lower score values on both scales indicated increased resistance (Online Resource 2b). Disease assessments were performed at different time-points from heading (Zadoks 50) to grain filling stage (Zadoks 85). Specifically, data were collected at three time-points in 2014 and two time-points in 2015 and 2016. The seven phenotypic datasets were referred to as: Field_2014_1, Field_2014_2, Field_2014_3, Field_2015_1, Field_2015_2, Field_2016_1and Field_2016_2.

### Genotyping

DNA of each accession was extracted according to the recommended DArT protocol (www.diversityarrays.com) and genotyped using the Genotyping-by-sequencing (GBS) Diversity Array Technology (DArTseq) platform (Li et al., 2015). A total of 56,306 raw silicoDArT markers were returned, of which 34,311 were polymorphic in the panel. Only markers positioned on the current DArTseq consensus map (i.e. 14,228 polymorphic markers) were selected and filtered based on 10% and 15% threshold for missing data for markers and accessions, respectively. Markers with a minor allele frequency (MAF) < 0.05 were also excluded. Additionally, three markers specific for known APR genes (*Yr18*, *Yr29* and *Yr46*) were included. In total, 13,934 high-quality markers and 292 accessions were retained and used for GWAS. These markers were ordered based on the genetic map positions in a high-resolution DArTseq consensus map (version 4.0), developed by Diversity Array Technology Inc., Canberra, ACT, Australia.

### Data analysis

Prior to analysis, seedling resistance scores (Stakman 0–4) were converted to the 0–9 scale where, 0 = immune and 9 = very susceptible, using a conversion table (Ziems et al. 2014). The IT was converted as follows: 0;,;n,;, 1 − , 1, 1 + , 2 − , 2, 2 + , 2 +  + , 3 − , 3, 3 + , 3 +  + and 4 were coded as 0, 0.5, 1, 2.5, 3, 3.5, 4, 5, 6, 6.5, 7, 8, 8.5 and 9, respectively. The accessions were grouped based on the phenotypic data (IT – Infection Type) of the seedlings; highly resistant (IT = 0‒3), intermediate (IT = 4‒6) and highly susceptible (IT = 7‒9). Accessions representing landraces, cultivars, breeding lines and the unclassified group displaying susceptible reactions at the seedling stage (IT = 7‒9) were selected (132 lines) for further analysis to identify lines carrying potentially novel APR genes.

Data analyses were performed using R (R Core Team 2019). To investigate groups of accessions within the Vavilov diversity panel, hierarchical cluster analysis was performed using passport data (including biological status and origin) and the YR disease response data. Squared Euclidean distances were used to calculate the dissimilarity matrix, and the tree was created according to Ward agglomerative clustering criterion. To compare the results across the seedling and adult plant experiments, correlation analyses were performed using mean disease severity (IT) scores. Principal component analysis (PCA) was performed using all datasets, and results were displayed as a biplot to investigate the correlation of disease responses across experiments.

### Identification of novel sources of APR

To identify accessions that most likely carried novel APR loci, disease response and PCR marker screening results were considered. First, the accessions which displayed high levels of susceptibility at the seedling stage (disease score ≥ 7) were selected assuming they lacked effective seedling resistance genes. Next, the accessions that carried one or more previously characterized APR gene(s) (*Yr18, Yr29*, *Yr46*, *Yr33*, *Yr39* and *Yr59*) were excluded. Finally, of the remaining accessions, those that displayed high levels of resistance at the adult plant stage were selected (*n* = 48).

### Genome-wide association studies

The genetic diversity and population structure of the Vavilov wheat diversity panel were previously described by Riaz et al. (2016a, b). GWAS was performed using a compressed mixed linear model (MLM) (Yu et al. 2006) implemented in the R package known as genome association and prediction integrated tool (GAPIT) (Lipka et al. 2012). To minimise spurious associations due to population structure and relatedness, the Q matrix was used as a fixed effect, and the K matrix was used to fit a random genetic effect (Gupta et al. 2014). To decrease the Type II error rate, a relaxed significance threshold for marker–trait associations (MTA) of –log_10_(*P*) > 3.0 was used, as previously employed in other GWAS studies examining the Vavilov panel (Riaz et al. 2018; Dinglasan et al. 2019). Markers detected in different assays, but in the same chromosomal position within a 1 cM window were considered as the same QTL. To confirm the independence of closely positioned QTL, the local LD (linkage disequilibrium) value for associated markers was calculated in R using the ‘genetics’ package (Warnes et al. 2012). For each significantly associated marker, the resistance allele was assigned based on the direction of the allele effect on the resistance score value (Riaz et al. 2018). For example, if the “1” marker allele had a negative effect (e.g. -1.2), it was considered the resistance allele and the “0” marker allele was considered the susceptibility allele.

### Comparison of QTL with previously reported Yr genes (QTL) and identification of putative candidate genes

To compare the position of the 47 QTL detected in this study with previously reported *Yr* genes and QTL, we projected the genetic positions on to the integrated consensus map developed by Maccaferri et al. (2015b) using the MapChart software version 3.2 (Voorrips, 2002) following the co-location strategy described in Ziems et al. (2014). Briefly, all the positions of QTL detected in this study (DArT wheat consensus map version 4) were positioned on the Maccaferri map using two approaches: 1) directly if the marker was present in both maps, or 2) projection using flanking markers in common across the maps. Once all QTL were positioned on the Maccaferri consensus map, the genomic regions were compared with previously reported *Yr* genes/QTL. The integrated consensus map includes markers from the 9 K (Cavanagh et al., 2013) and 90 K (Wang et al., 2014b) consensus maps, the tetraploid consensus map (Maccaferri et al., 2015a), the Synthetic × Opata DH GBS map (Saintenac et al. 2013), the Diversity Array Technology (DArT) integrated map (http://www.diversityarrays.com/search/node/wheat%20DArT%20map), SSR consensus map (Somers et al. 2004) and the Synthetic × Opata ITMI BARC SSR map (Song et al. 2005). In addition, recently reported genes were also projected onto the integrated map. All QTL regions were compared with the genes/QTL reported by Chen (2017). QTL were considered novel when they were positioned more than 10 cM away from a previously reported gene or QTL. The sequences for flanking markers associated with QTL identified in this study were BLAST searched against the Ensemble Plant genome-centric portal to find the physical position based on the wheat genome assembly IWGSC Ref-Sequence v1.1 (IWGSC 2018). For five DArTSeq markers (1,255,550, 981,525, 1,236,960, 3,026,338, 1,159,261), the 10 + genome assembly browsers at GrainGenes (http://www.graingenes.org) were used to retrieve the physical position.

### Assigning and stacking of alleles associated with novel APR QTL

Seven newly discovered APR QTL were further analysed to determine their effect on disease response. To remove the masking effect of major resistance genes, only accessions displaying susceptibility at the seedling stage (i.e. IT scores > 7) were selected for these analyses. The difference between the mean disease responses for groups of accessions carrying different combinations of resistance alleles was tested for significance based on Tukey’s test for multiple comparisons with a family-wise error rate of 5%. To investigate the trend between disease response and the accumulation of resistance alleles for the seven APR QTL, a relative disease index (YRi) was calculated across field trials according to:

$$YRi=\sum_{k}^{n}\frac{individual Dis. Score [k]}{mean Dis.Score [k]}$$

where YRi is the disease index of a line, in experiment k in relation to the population mean over n field experiments. A disease index below 0 reflects a high level of resistance to YR across the trials, whereas values above 0 imply susceptibility.

## Results

### Analysis of stripe rust response at seedling and adult plant stages

**Fig. 1:**
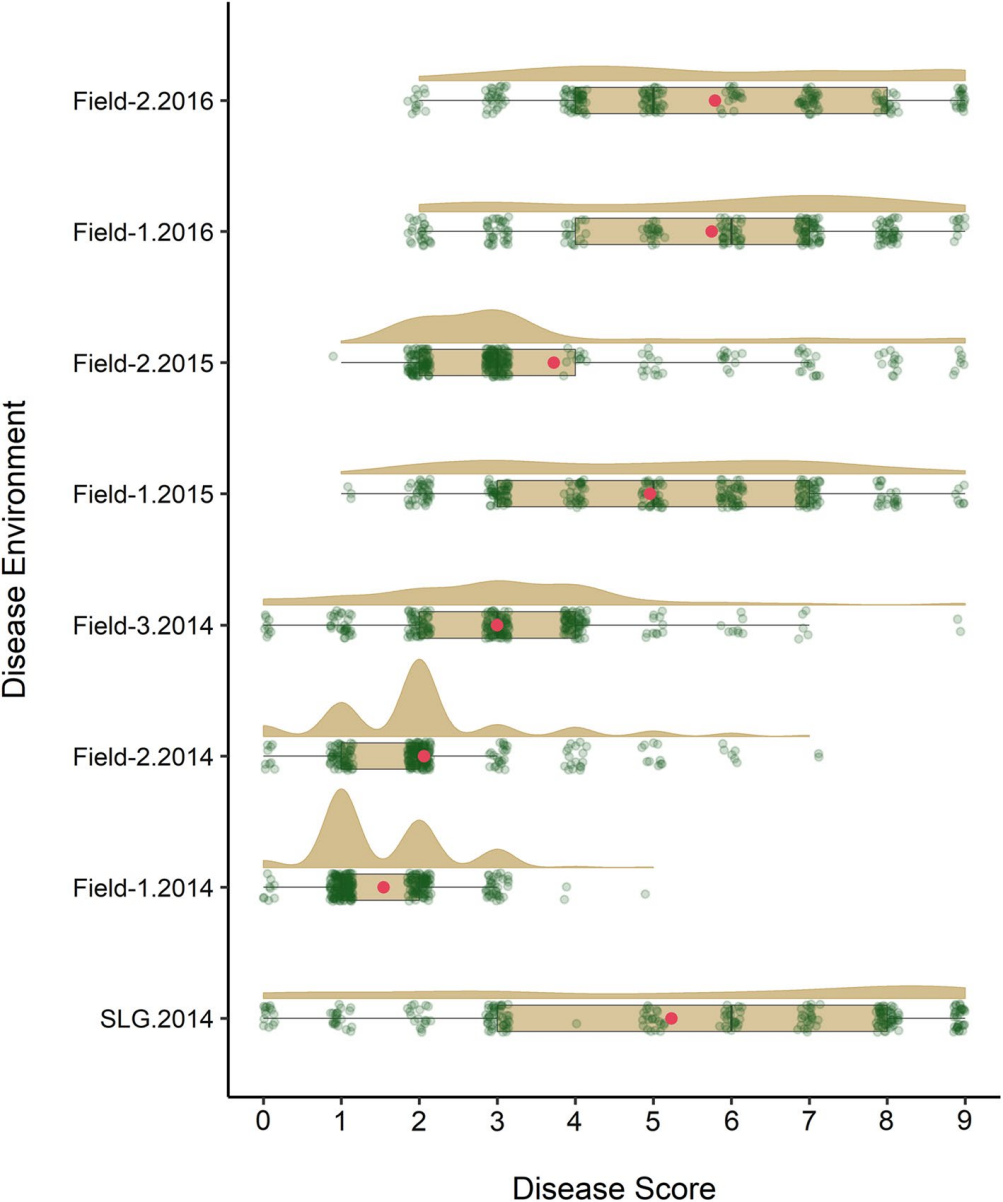
Distribution of YR response in the Vavilov wheat diversity panel evaluated in seedling (SLG) and adult plant field environments in 2014, 2015 and 2016. The boxplot shows the median value with upper and lower quartile range and overlaid green dots are the jittered row data points, while the red dot represents the mean disease score; the half violin plot represents the estimated density distribution

In total, 292 accessions were tested for their IT at the seedling stage and the results revealed that 39% of the accessions showed highly resistant reactions (IT = 0–3), 16% moderately resistant reactions (IT = 4–6) and 45% were susceptible (IT = 7–9). Most accessions displaying susceptibility were of unknown origin (*n* = 50; Online Resource 1), followed by accessions from Russia (*n* = 27), India (*n* = 17) and Pakistan (*n* = 11). In total, 132 lines displayed susceptible reactions to YR at the seedling stage, and therefore, these accessions were considered potential candidates for carrying uncharacterised APR genes. Under field conditions, the accessions displayed a wide range of responses across the three years (Fig. 1). In 2014 and 2015, more than 80% of the tested lines (*n* = 284) displayed resistant to moderately resistant reactions, whereas in 2016, only 58% of the tested lines (*n* = 247) showed resistant reactions of which 43% displayed intermediate resistant phenotypes (Fig. 1).

Weak positive correlations were observed between seedling and most field-based assessments; for example, Field_2014_3 (*r* = *0.33, P* < *0.001*) and Field_2015_2 (*r* = *0.30, P* < *0.001*). The seedling disease response showed a moderate correlation with Field_2016_2 *(r* = *0.53, P* < *0.001*). Conversely, stronger correlations were observed between the field assessments; for example, Field_2015_1 and Field_2016_2 (*r* = *0.81, P* < *0.001*). In Online Resource 3, the accessions displayed in black are seedling-susceptible and lack known genes, thus representing accessions that could carry uncharacterised APR.

### Cluster analysis using phenotype and passport data

**Fig. 2:**
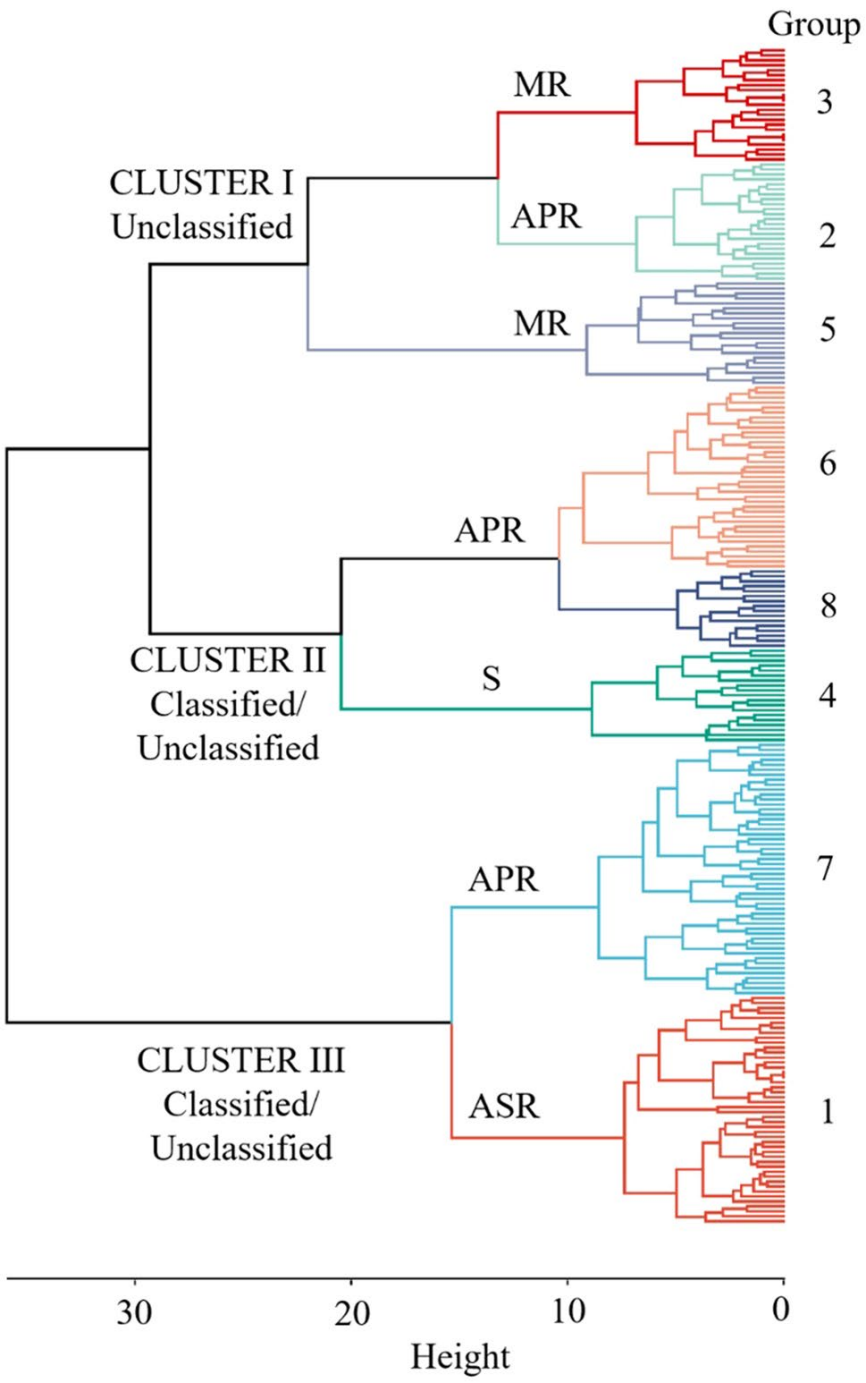
Ward’s hierarchical agglomerative clustering of 238 Vavilov wheat lines generated from biological status (Unclassified, Classified–Breeding line, Cultivar, Landrace), origin of collection and stripe rust disease response in the glasshouse and field experiments. Dendrogram height (0–35) is represented by the *horizontal line*. Eight subgroups were delineated (height 10) which represent level of stripe rust resistance

Ward’s hierarchical agglomerative cluster analysis was performed for 238 wheat accessions using trait data, including biological status (Unclassified, Classified – Breeding line, Cultivar, Landrace), origin of collection and YR disease response in the glasshouse and field experiments. The analysis revealed three major clusters divided into eight subgroups (Fig. 2 and Online Resource 1). Cluster I comprised accessions belonging only to the unclassified group (i.e. unknown geographical information and unknown biological status). All the accessions in this cluster were grouped under the APR and moderate resistance (MR) category (Subgroups 2, 3 and 5). The two other major clusters (Cluster II and III) comprised accessions belonging to a combination of classified and unclassified groups. Cluster II comprised accessions carrying APR (Subgroups 6 and 8) and accessions that were generally susceptible (Subgroup 4). Accessions in Subgroup 6 were more resistant and stable across the years compared to accessions belonging to Subgroup 8. Cluster III comprised accessions carrying mainly APR (Subgroup 7) and ASR (Subgroup 1).

### Identification of novel sources of APR

**Fig. 3:**
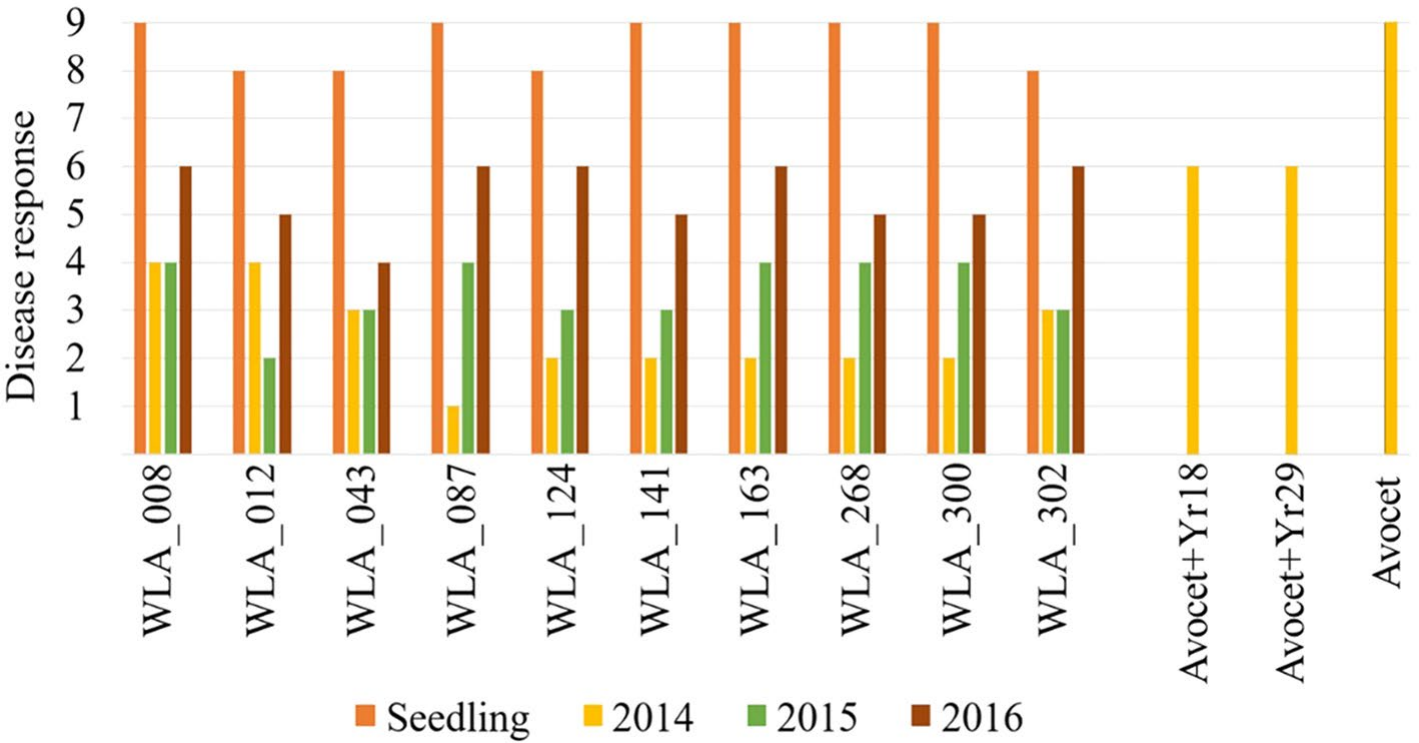
Seedling and adult plant responses to stripe rust for the 10 lines most promising lines carrying potentially novel sources of adult plant resistance (APR). Orange bars represent the disease response at the seedling stage, whereas yellow, green and brown represent disease responses at the adult plant stage in 2014, 2015 and 2016, respectively. Avocet and the APR near-isogenic lines (Avocet + *Yr18* and Avocet + *Yr29*) were evaluated at the adult stage in 2014 only

Based on the results from YR assessments and PCR marker screening, 48 lines were deemed to carry potentially novel sources of APR to YR (Online resource 3). This included lines classified as unknown origin (*n* = 18), landraces (*n* = 14), cultivars (*n* = 10) and breeding lines (*n* = 6). The landraces were mainly from India (*n* = 3), Spain (*n* = 2), Russia (*n* = 2), and Kazakhstan (*n* = 2), although landraces from Pakistan, Turkey, Azerbaijan, Tajikistan, and Armenia were also included in the set. Most cultivars (*n* = 8) and breeding lines (*n* = 5) that carried potentially novel sources of APR were from Russia. The 10 most promising lines were selected for crossing to elite cultivars. These showed a high degree of susceptibility at the seedling stage and intermediate levels of resistance at the adult plant stage (Fig. 3). The majority of these lines were from the unclassified group (*n* = 6), followed by a landrace from Pakistan (WLA–043), a breeding line from Russia (WLA–302) and two cultivars from Chile (WLA–300) and Russia (WLA–087).

### Marker–trait associations

A total of 68 significant marker–trait associations were detected (-log_10_ (*p value*) > 3) which represented 47 different QTL regions (Table 1, Online Resource 4). Fifty-seven markers were associated with resistance at the adult plant stage, and many (*n* = 26) were detected only under the environmental conditions experienced in 2014. Nine markers were associated with resistance at the seedling stage, while only one marker was detected at both seedling and adult plant stage. Based on the chromosomal position and LD between adjacent markers, the QTL represented 41 APR loci and 6 seedling resistance loci. Comparison of these QTL regions with the previously reported *Yr* genes and QTL identified seven genomic regions as potentially novel QTL associated with YR resistance at the adult plant stage (Online Resource 5, 6). Notably, 14 QTL overlapped with the positions of previously reported *Yr* genes: *Yr29*, *Yr5*, *Yr43*, *Yr57*, *Yr30*, *Yr51*, *Yr48*, *Yr47*, *Yr35*, *Yr36*, *Yrxy1*, *Yr59*, *Yr52* and *YrYL* on chromosome 1B, 2B, 3B, 4A, 5B, 6B, 7A, 7B and 7D, suggesting they could be linked to, or represent, those known resistance factors (Online resource 6).

**Table 1:** Summary of the stripe rust resistance QTL identified at both the seedling and adult plant stage in the Vavilov wheat diversity panel

QTL name	Peak Marker ^a^	Chr	Position (cM)^b^	Position (Mb)^c^	Phenotypic dataset(s)^d^	− log_10_(*P*)	R. allele ^e^	Effect on trait	Gene/QTL	References
*qNV.Yr-1B-1*	1,219,437	1B	241.91	660.6	Field_2_2016	3.14	1	− 0.2	Previously reported	Maccaferri et al. 2015a, b
*qNV.Yr-1B-2*	1,119,286	1B	269.26	677.4	Field_2_2016	3.34	0	− 0.2	*Yr29*	Lan et al. 2014,
										Maccaferri et al. 2015a, b
*qNV.Yr-1D-1*	1,862,252	1D	29.72	21	Field_3_2014	3.17	1	− 0.8	New	
*qNV.Yr-1D-2*	1,229,892	1D	47.7	35.7	Field_2_2016	3.23	1	− 0.22	Previously reported	Maccaferri et al. 2015a, b
*qNV.Yr-2A.1*	2,295,954	2A	9.93	12.2	Field_1_2015	3.2	1	0.05	Previously reported	Maccaferri et al., 2015a, b
*qNV.Yr-2A.2*	1,255,550	2A	24.56	33.6	Seedling	3.5	1	0.05	*Yr56*	Bansal et al. 2014
										Basnet et al. 2014a, b
*qNV.Yr-2A.3*	1,093,102	2A	46.78	49.5	Field_2_2015	3.78	1	− 0.2	Previously reported	Naruoka et al. 2015
*qNV.Yr-2A.4*	1,122,504	2A	60.52	87	Field_1_2015	3.59	1	0.08	Previously reported	Bulli et al. 2016
*qNV.Yr-2A.5*	1,862,406	2A	68.57	256.2	Field_2_2015	3.45	1	0.11	New	
*qNV.Yr-2A.6*	1,183,380	2A	124.25	761.1	Field_2_2014	3.6	0	0.23	Previously reported	Dedryver et al. 2009
					Field_3_2014	3.76		0.79		
*qNV.Yr-2B.1*	2,289,349	2B	78.31	583.7	Field_2_2016	3.45	1	0.29	*Yr5*	Godoy et al. 2017
*qNV.Yr-2B.2*	1,235,357		88.54	763	Field _2_2016	3.19	0	0.37	*Yr43*	Maccaferri et al. 2015a, b,
	1,233,396		88.92	763.4	Seedling	3.43	0	− 0.51		Xu et al. 2013
	3,064,531		88.98	763.5	Seedling	4.11	0	0.07		
	2,322,232		89.52	765.6	Seedling	3.06	1	0.001		
	981,525		89.52	764.6	Seedling	3.23	1	− 0.07		
	1,046,333		89.52	759.7	Seedling	3.21	1	− 0.06		
	2,322,248		89.88	766.2	Field_1_2015	3.45	1	0.1		
*qNV.Yr-2B.3*	1,236,960	2B	107.02	783.9	Field_2_2015	4.33	0	− 0.33	Previously reported	Crossa et al. 2007,
	1,117,771			784.4	Field _2_2015	4.41	0	− 0.4		Buerstmayr et al. 2014
					Field_2_2014	3.44		1.31		
	1,337,290			790.6	Field_2_2014	4.11	1	− 0.57		
	1,296,890			790.6	Field_2_2015	3.01	0	− 0.12		
	1,297,955			790.9	Field_2_2015	3.63	0	− 0.6		
	4,663,965			794.9	Field_2_2015	4.15	0	− 1.08		
	1,298,829			800.1	Field_2_2015	3.24	0	− 0.41		
*qNV.Yr-2D.1*	2,246,033	2D	70.85	207.1	Field_2_2014	3.35	1	0.97	Previously reported	Mallard et al. 2005,
	2,338,436			337.4	Field_2_2015	3.08	1	0.15		Ren et al. 2012a, b
*qNV.Yr-2D.2*	2,243,695	2D	136.45	619.6	Seedling	3.04	0	0.45	Previously reported	Suenaga et al. 2003,
										Melichar et al. 2008
*qNV.Yr-3A.1*	1,123,104	3A	44.75	170.1	Seedling	3.22	1	0.33	Previously reported	Zegeye et al. 2014
*qNV.Yr-3A.2*	2,253,468	3A	49.23	574.3	Field_1_2015	3.41	0	0.06	Previously reported	Pasam et al. 2017
*qNV.Yr-3A.3*	2,295,584	3A	53.26	600.3	Field_3_2014	3.09	1	1.5	Previously reported	Hou et al. 2015
*qNV.Yr-3A.4*	1,116,501	3A	109.54	100.4	Field_2_2015	3.23	0	− 0.11	Previously reported	Rosewarne et al. 2013
*qNV.Yr-3A.5*	1,237,203	3A	142.13	738.9	Field_1_2014	4.38	0	0.6	New	
*qNV.Yr-3B*	2,307,351	3B	4.97	5.3	Field_1_2016	4.28	0	− 0.23	*Yr57*	Lan et al. 2014,
					Field_2_2016	3.03		− 0.13	*Yr30*	Basnet et al. 2014a
	1,239,212		5.25	5.3	Field_1_2016	3.83	0	− 0.02		
					Field_2_2016	3.24		− 0.006		
					Seedling	3.27		− 0.07		
*qNV.Yr-3D.1*	992,483	3D	59.49	374	Field_1_2014	3.88	1	− 0.41	New	
*qNV.Yr-3D.2*	1,228,106	3D	123.84	582.5	Field_2_2014	3.45	0	3.73	Previously reported	Basnet et al. 2014a
*qNV.Yr-4A.1*	1,120,960	4A	96.08	668.4	Field_2_2014	3.76	1	1.02	Previously reported	Bulli et al.^2016^,
										Zegeye et al. 2014
*qNV.Yr-4A.2*	1,131,408	4A	117.26	252.7	Field_2-2016	3.79	0	0.42	Previously reported	Vazquez et al. 2012
*qNV.Yr-4D.1*	3,021,792	4D	43.44	158.4	Field_2_2016	3.97	1	− 0.38	*Yr46*	Herrera-Foessel et al. 2011
					Field_2_2014	3.74		0.3		
					Field_1_2016	3.4		− 0.07		
*qNV.Yr-4D.2*	1,117,919	4D	54.74	439.3	Field_1_2014	3.08	1	1.19	Previously reported	Ren et al. 2012a, b
*qNV.Yr-5A*	1,127,003	5A	58.39	524.8	Seedling	4.8	0	− 0.03	Previously reported	Manickavelu et al. 2016,
										Lan et al. 2014
*qNV.Yr-5B.1*	1,088,238	5B	12.41	15.3	Field_2_2015	3.26	0	NA	*Yr47*	Bansal et al. 2013
*qNV.Yr-5B.2*	1,125,706	5B	22.69	42.3	Field_2_2016	3.07	1	− 0.01	Previously reported	Hao et al.^2011^
*qNV.Yr-5B.3*	1,104,944	5B	27.83	54.2	Field_2_2015	3.07	0	0.04	Previously reported	Yang et al. 2013,
	1,128,792		29.32	53	Field_2_2015	3.03	0	0.01		Lu et al. 2014
*qNV.Yr-5B.4*	1,107,669	5B	46.54	590	Field_2_2014	3.15	1	0.44	Previously reported	Lu et al. 2009,
					Field_3_2014	3.41		1.64		Vazquez et al. 2015
*qNV.Yr-5D*	991,465	5D	58.61	424	Field_1_2014	3.62	0	0.14	New	
*qNV.Yr-6A*	3,022,417	6A	100.76	616.3	Field_2_2016	3.61	0	− 0.01	Previously reported	Vazquez et al. 2012,
										Bulli et al. 2016
*qNV.Yr-6B.1*	1,302,727	6B	2.41	9.8	Field_2_2014	3.64	0	− 1.78	*Yr35*	Prins et al. 2011,
	1,097,180		2.41	0.3	Field_1_2015	3.19	0	− 0.2		Bansal et al. 2013
	1,121,581		4.36	18	Field_1_2014	3.89	1	1.06		
*qNV.Yr-6B.2*	1,229,248	6B	10.89	20.7	Field_3_2014	3.56	0	8.01	Previously reported	Prins et al. 2011, Bansal et al. 2013
*qNV.Yr-6B.3*	2,290,856	6B	23.19	117.1	Field_1_2015	3.63	1	0.12	*Yr36*	Pasam et al. 2017,
										Santra et al. 2008
*qNV.Yr-6B.4*	1,058,394	6B	35.34	576.5	Field_2_2016	3.55	0	0.09	Previously reported	Klarquist et al. 2016
*qNV.Yr-6B.5*	2,293,763	6B	79.73	710.6	Field_3_2014	3.07	1	− 3.44	Previously reported	Basnet et al. 2014a,
	1,109,194		82.95	715.8	Field_1_2015	3.19	0	− 0.37		Rosewarne et al. 2013
					Field_2_2016	3.28		− 0.31		
*qNV.Yr-7A.1*	1,244,188	7A	96.07	648.3	Field_2_2015	3.34	0	0.15	*Yrxy1*	Zhou et al. 2011,
										Singh et al., 2014a, b,
										Zegeye et al. 2014
*qNV.Yr-7A.2*	1,090,077	7A	149.56	712.3	Field_2_2015	3.85	1	− 0.04	Previously reported	Rosewarne et al. 2013
										Vazquez et al. 2012
*qNV.Yr-7B.1*	1,126,816	7B	20.97	170.5	Field_2_2014	3.26	1	2.31	New	
*qNV.Yr-7B.2*	1,059,624	7B	38.66	138.7	Field_2_2014	3.23	1	0.12	Previously reported	Suenaga et al. 2003
	1,259,637		38.65	129.5	Field_1_2014	3.17		− 0.01		
*qNV.Yr-7B.3*	1,298,605	7B	58.63	607.6	Field_3_2014	3.21	0	− 0.11	Previously reported	Pasam et al. 2017
	3,026,338		59.74	614.1	Field_3_2014	3.33	0	− 2.98		
	1,159,261		60.75	614.1	Field_1_2014	3.03	0	0.01		
					Field_3_2014	3.24		0.6		
*qNV.Yr-7B.4*	1,066,421	7B	118.47	718.1	Field_1_2014	3.36	1	0.14	*Yr52*	Ren et al. 2012a, b,
									*Yr59*	Zhou et al.^2014^
*qNV.Yr-7D.1*	1,105,401	7D	56.58	65.9	Field_2_2015	3.3	1	− 0.27	*YrYL*	Wu et al. 2016
*qNV.Yr-7D.2*	2,303,363	7D	72.12	103.6	Field_1_2016	3.03	1	− 0.04	New	

^a^Most strongly associated marker for the QTL

^b^Chromosome position (cM) of the QTL on the DArTseq wheat consensus map (v 4.0)

^c^Chromosome position (Mb) of the QTL on the physical map (Ref-Sequence v1.1 (IWGSC 2018), 10 + genome assembly browsers—GrainGenes)

^d^The dataset for which the QTL was detected

^e^Allele associated with resistance

Six QTL regions defined by 10 markers (2A, 2B, 2D, 3A, 3B and 5A) were associated with seedling resistance. Of these QTL, only four were detected at the seedling stage, while the remaining two QTL (*qNV.Yr-2B.2* and *qNV.Yr-3B*) were detected at both seedling and adult plant growth stages. The QTL *qNV.Yr-2B.2* included seven markers within the 88.5–89.9 cM region, which were associated with resistance across test environments (i.e. seedling, field trials in 2015 and 2016). It also co-located with the seedling resistance gene *Yr43*. The remaining three QTL were detected only in one adult plant environment (besides the seedling stage). The QTL with the strongest association was *qNV.Yr-3B* (-log_10_ *P* ≥ 4) with larger marker effects on disease response (-0.23). This QTL co-located with partial resistance (and pleiotropic) gene *Yr30* and the seedling resistance gene *Yr57*, which provides resistance against the Australian pathotype 134E16 A + Yr17 + Yr27. However, due to the narrow genomic interval between these genes on chromosome 3B, we were unable to determine whether *Yr30*, *Yr57* or both were present in this QTL.

A total of 41 QTL (57 markers) were considered “APR QTL” and were significantly associated with IT in one or multiple environments (Table 1). A total of 26, 23 and 16 markers were detected in 2014, 2015 and 2016, respectively, and clustered into different QTL regions. Comparison of the detected loci with the previously reported *Yr* genes and QTL (using the integrated map from Maccaferri et al. 2015b) revealed that 41 genomic regions likely represented previously reported genes and/or QTL (Table 1, Online Resource 5, 6 and 7). Interestingly, genomic regions harbouring the HTAP resistance genes *Yr36*, *Yr59* and *Yr52* were detected in the 2014 and 2015 field environments, whereas APR genes *Yr29* and *Yr30* were detected in 2016. Notably, high average maximum temperatures (28.5 °C ‒ 31.4 °C) were experienced during the YR assessment periods in 2014 and 2015 compared to 2016.

Seven QTL (chromosomes 1D, 2A, 3A, 3D, 5D, 7B and 7D) were detected at the adult plant stage and appeared to be novel (Table 1). The QTL *qNV-Yr-7D.2* was detected under environmental conditions in 2016, whereas all other QTL in the D genome were detected in 2014. The QTL *qNV.Yr-2A.5* and *qNV.Yr-5D* were significant in multiple field environments, while *qNV.Yr-1D-1*, *qNV.Yr-3A.5*, *qNV.Yr-3D.1*, *qNV.Yr-7B.1*, and *QYr.uq-7D.2* were detected only in a single field environment.

### Allele stacking effects and geographical distribution of the novel APR QTL

**Fig. 4:**
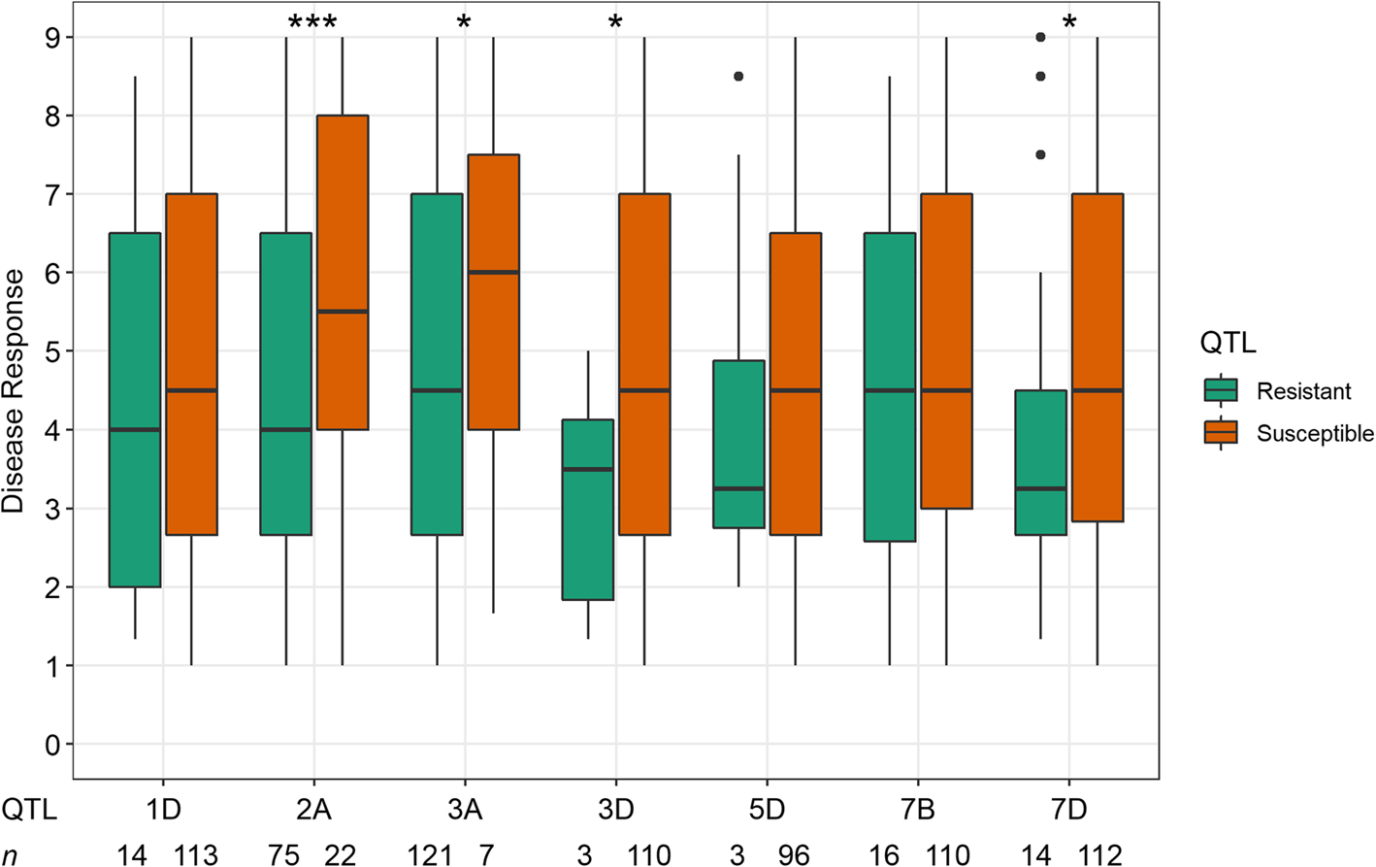
Phenotypic effect of the novel QTL for adult plant resistance to stripe rust in the Vavilov wheat diversity panel. Colour fill indicates the presence (green) or absence (orange) of the resistance allele for the corresponding QTL, wherein QTL in chromosome 1D = *q.NV.Yr-1D.1*, 2A = *q.NV.Yr-2A.5*, 3A = *q.NV.Yr-3A.5*, 3D = *q.NV.Yr-3D.1*, 5D = *q.NV.Yr-5D*, 7B = *q.NV.Yr.7B.1*, and 7D = *q.NV.Yr-7D.2*. The horizontal line in the boxplot represents the median value, and “***” and “*” show the significant level at *P* < 0.001 and 0.05, respectively

**Fig. 5:**
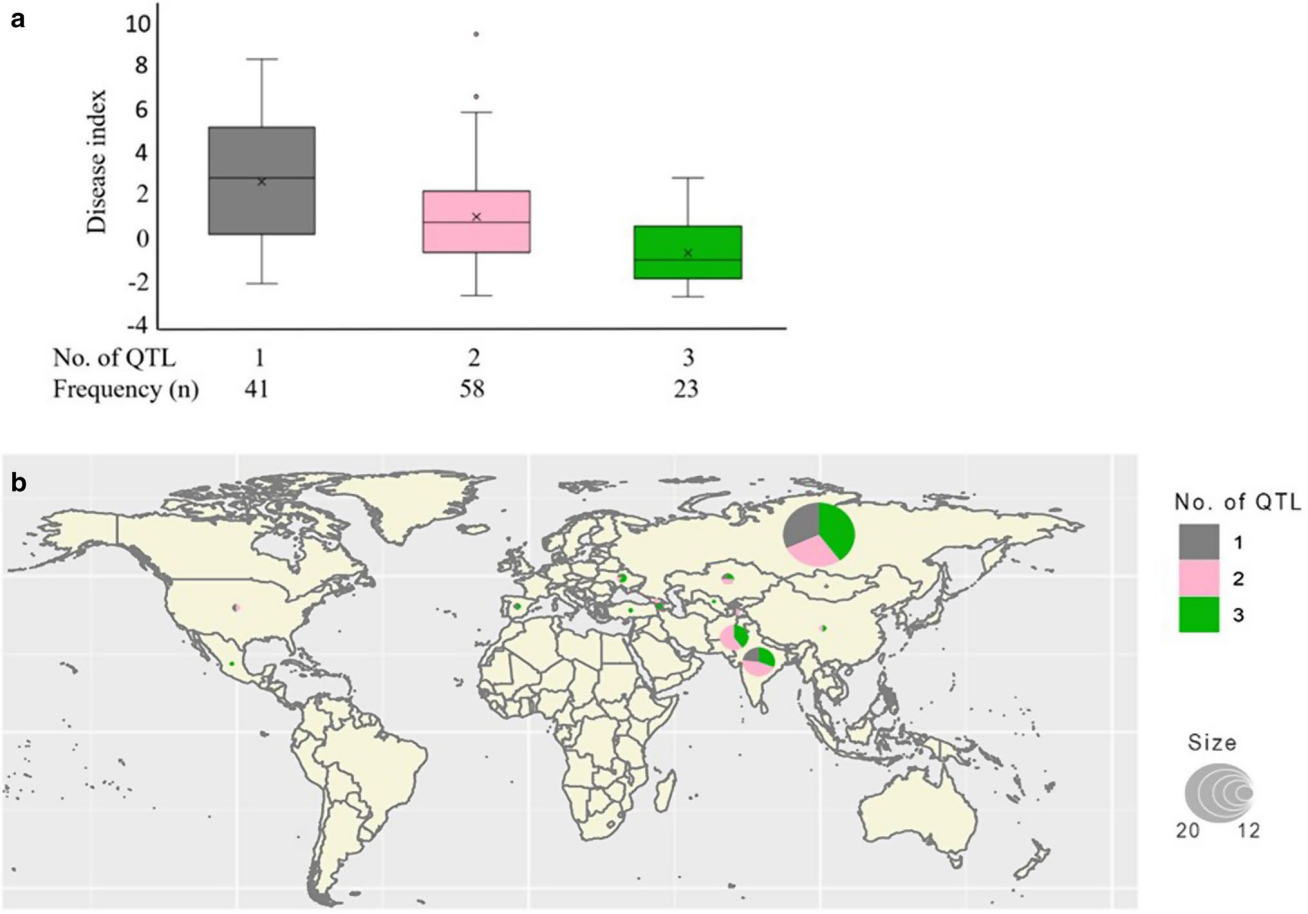
The stacking effect for novel adult plant resistance (APR) alleles and their geographical distribution according to origin of the Vavilov accessions. **a** The effect of the number of novel alleles on resistance response to APR to stripe rust in the Vavilov wheat diversity panel. The disease index is calculated using field-based disease response data. The frequency of lines carrying 1, 2 or 3 favourable alleles is also presented. **b** Geographic distribution of the novel resistance alleles in the diversity panel. The colour gradients represent the number of QTL present in accessions originating from each country

The seven novel APR QTL were investigated for their potential additive effect to reduce disease severity. A total of 132 accessions that were seedling susceptible were selected for the analysis. To further verify allelic effects in the subset, the mean resistance level of lines carrying alleles associated with resistance was compared with those carrying alleles for susceptibility at each locus. Significant allelic effects were verified through t-tests for *qNV.Yr-2A.5*, *qNV.Yr-3A.5*, *qNV.Yr-3D.1*, and *qNV.Yr-7D.2*, whereas no significant effects were detected for *qNV.Yr-1D-1*, *qNV.Yr-5D* and *qNV.Yr-7B.1* (Fig. 4). To investigate the accumulation effect of the seven novel APR loci for YR resistance, lines were grouped based on the number of QTL present in each line. A clear decreasing trend in the field relative disease index was observed as the number of resistance alleles increased from one to three (Fig. 5a). None of the Vavilov lines combined more than three resistance alleles. Notably, most of the lines carrying three novel resistance loci (*n* = 23) showed a disease index lower than zero. The novel alleles occurred singly or in combination in accessions from India, Kazakhstan, Ukraine, and Russia (Fig. 5b). Further, countries from central Asia to west Asia such as Uzbekistan, Tajikistan, Armenia, and Georgia also carried combinations of 2–3 novel alleles.

## Discussion

### Vavilov wheat accessions offer diverse sources of stripe rust resistance

In this study, the Vavilov wheat accessions displayed a wide range of YR responses in both seedling and adult plant stages across multiple environments. At the seedling stage, about 55% of the accessions displayed high levels of resistance (39%, IT = 0–3) to intermediate levels of resistance (16%, IT = 4–6). Based on field assessments in 2014, 83% of the lines also showed resistance at the adult plant stage, indicating the presence of strong seedling resistance or combination of effective seedling and APR to YR. Alternatively, these resistant accessions could carry multiple APR genes, as the presence of just 2 or 3 minor genes can confer “near-immune” levels of resistance (Bariana et al. 2010; Singh et al. 2014b). Some genes are known to have a synergistic effect on disease resistance, such as *Lr34*/*Yr18* (German and Kolmer 1992), which directly contributes resistance to leaf rust, YR and powdery mildew (Rinaldo et al. 2017), while also contributing resistance to stem rust due to additive and epistatic interactions (Hiebert et al. 2016). These types of genes are more valuable when breeding for rust resistance because they are less likely to be selected and deployed alone, and some genes can enhance resistance levels to more than one economically important disease.

A total of 45% of the accessions (*n* = 132) displayed susceptibility at the seedling stage, but resistance at adult growth stages. Considering the diversity of the germplasm, it suggests a high frequency of multiple APR genes. Despite using the same pathotype each year, the correlation between disease response varied across the three years of field evaluations. This highlights the role of genotype × environment interactions influencing the expression of resistance. The low temperature and high rainfall in 2016 compared to 2014 and 2015 likely favoured disease development and could be the reason for increased susceptibility in 2016. Although, this does not rule out other factors related to seasonal variation such as differences in physiological development across seasons (Wang et al. 2014) and abiotic stress (Guo et al. 2008). Such effects are well described for the *Lr34*/*Yr18* gene, where studies have found that gene expression is highly influenced by the genetic background, environmental factors and level of infection (Risk et al. 2012).

Screening for known APR genes (*Yr18*, *Yr29*, *Yr46*, *Yr33*, *Yr39*, and *Yr59*) using linked molecular markers revealed some interesting relationships when accession passport information was explored. For instance, the presence of one or more gene combinations of *Yr59* and *Yr46* in almost all accessions from India and Pakistan suggests that these APR genes are common in germplasm originating from these regions. A total of the 88 (65%) lines that displayed strong resistant responses in both seedling and adult plant stages were classified as landraces. Previous studies have also found that wheat landraces offer a rich source of disease resistance loci (Tala et al. 2011; Pasam et al. 2017).

By combining the field screening data with results from marker screening for known APR genes, we identified accessions that carried potentially novel resistance alleles. Interestingly, a number of lines originating from European and Asian countries such as Russia, Kazakhstan, Armenia, Ukraine, Tajikistan, Spain, Uzbekistan, Turkey, Mongolia, and China showed an absence of known APR genes and clustered in the APR category, which suggests they likely carry novel resistance alleles. The top 10 lines deemed to carry potentially novel sources of APR were from Russia, Pakistan, Chile, and the group of unknown origin. The Russian accessions WLA–302 (1978) and WLA–087 (1990) displayed intermediate resistance across the three years of evaluation. The landrace WLA–043 from Pakistan collected in 1936 was found to be a good source of APR to YR across the years. Pakistan is a diversity hotspot for wheat due to its wide agroecological zones, where many landraces that are genetically diverse with rare alleles have been found, particularly in the Himalayan region (Hirano et al. 2008). Evaluation of 5,700 accessions from USDA‒ARS National Small Grain Collection (NSGS) showed that Chile is another important geographic centre of rust resistance with the highest number of accessions (28%) for stem rust resistance determined at the adult plant stage (Bonman et al. 2007). In this study, the cultivar WLA–300 collected from Chile in 1963 was identified as one of the best accessions for APR to YR. The top 10 accessions identified in this study should be prioritised for bi-parental mapping studies to characterise the underlying resistance loci.

### GWAS reveals 47 genomic regions associated with resistance

This study identified 68 DArTseq markers, representing 47 QTL regions, that were significantly (*P* < 0.001) associated with seedling and adult plant resistance to YR. Notably, a larger number of marker–trait associations (*n* = 26) were detected under field conditions in 2014, which represented 21 QTL regions. In contrast, the markers detected in 2016 (*n* = 15) represented 14 different genomic regions and 13 of them were unique to the field environment in 2016. Therefore, the field conditions experienced in 2016 resulted in the detection of many loci that were either not expressed or weakly expressed in the 2014 and 2015 field environments because GWAS was unable to detect them. This highlights the importance of testing material for rust resistance across multiple environments.

Among the 6 QTL detected at the seedling stage, a major haplotype block comprising seven markers was identified on chromosome 2B (*qNV.Yr-2B.2*). These markers were significantly associated with resistance at seedling, adult plant or both stages, suggesting the possible presence of more than one locus within this region. Previously, four different genes (*Yr5*, *Yr44*, *Yr53* and *Yr43*) have all been mapped in this region within 35.1 cM (Xu et al. 2013). Based on the positions of key markers, *Yr43* is most likely the underlying gene for *qNV.Yr-2B.2*. It should be noted that virulence for *Yr43* and *Yr44* occurs in many *Pst* races that emerged from 2000 onwards in the USA (Chen 2005; Wan and Chen 2014), while *Yr5* and *Yr53* have remained effective against all races. Therefore, it is important to study this QTL in detail to understand the specific gene/genes which confer resistance to the Australian pathotype 134E16 A + Yr17 + Yr27. Such information would be very useful for breeding programmes.

Three well-characterised APR multi-resistance loci were detected in this study, including *Lr46/Yr29/Sr58/Pm39/Ltn2* on chromosome 1BS, *Sr2/Yr30/Lr27/Pm* on 3BL and *Lr67/Yr46/Pm46/Sr55/Ltn3* on 4DS. These are the most commonly introduced and selected genes in global wheat breeding programmes. Interestingly, effects associated with these genes were detected in the 2016 field environment that experienced relatively lower temperatures and higher rainfall, in comparison to the 2014 and 2015 seasons. Apart from these genes, only a few QTL were detected in 2016 compared to the other years. The QTL (*QYr.ucw-1B* and *QYr.ucw-1D*) reported by Maccaferri et al. (2015a) and QTL (*QYrst.orr-4A* and *QYrst.orr-6A*) reported by Vazquez et al. (2012) were also detected in the present study in 2016. The resistance conferred by these QTL may be further studied in detail by analysing two seedling-susceptible landraces from Armenia (WLA–249) and Russia (WLA–315). Further, the HTAP resistance genes such as *Yr36* (Uauy et al. 2005), *Yr52* (Ren et al. 2012a, b) and *Yr59* (Zhou et al. 2014) coincided with QTL detected in 2014 and 2015 field environments which were much warmer than 2016, which further suggests these regions could be underpinned by HTAP resistance loci.

Other important QTL detected in this study were *qNV.Yr-3B* and *qNV.Yr-7B.4* (Table 1). The first QTL, *qNV.Yr-3B,* was detected at the seedling stage; however, this QTL was also detected at the adult stage under field conditions in 2016. *qNV.Yr-3B* co-located with seedling resistance gene *Yr57* and adult plant pleiotropic gene *Yr30*. *Yr57* is a broadly effective against Australian pre-and post-2000 *Pst* pathotypes 104 E137A + , 108E141A + , 110 E143A + , 134 E16A + , 134 E16A + Yr17 + , 134 E16A + Yr17 + Yr27 + and 150 E16A + (Randawa 2015), whereas *Yr30* is closely linked to *Sr2* and confers partial resistance to YR. These two genes are closely linked (< 5 cM apart), which could be exploited for gene pyramiding (Randawa 2015). The second QTL, *qNV.Yr-7B.4,* were represented by DArT marker 1,066,421 in the field in 2014. Notably, this region on chromosome 7B harbours two HTAP genes, *Yr52* (Ren et al. 2012a, b) and *Yr59* (Zhou et al. 2014), and two temporally designated genes *YrC591* (Li et al 2008) and *YrZH84* (Li et al. 2006). *Yr52* confers HTAP resistance, which is highly consistent and partial in nature, making it an attractive target for breeding programmes targeting durable rust resistance (Ren et al, 2012a, b). *Yr59* is another important HTAP resistance gene, which is linked to *YrZH84* (Zhou et al. 2014) and is effective against all known North American *Pst* races; thus, it is considered highly valuable for resistance breeding.

### Novel QTL for adult plant resistance

Seven new QTL for YR resistance were identified on chromosomes 1D, 2A, 3A, 3D, 5D, 7B and 7D. These genomic regions were associated with different levels of resistance across the three years of field assessments. *qNV.Yr-2A.5* (chromosome 2A) and *qNV.Yr-5D* (chromosome 5D) were associated with resistance in 2014 and 2015, while other QTL were detected only in a single environment. Therefore, these QTL may be environment-specific or convey smaller effects. Notably, the majority of novel QTL detected in this study were located in the D-genome. *Aegilops tauschii*, the D-genome progenitor of hexaploid wheat, is considered a valuable source of YR resistance (Liu et al. 2013). However, until now, only a few resistance loci have been identified in the D-genome. For instance, *Yr28* (Singh et al. 2000), slow rusting APR gene *Yr46* (Herrera foessel. 2011) and *YrAs2388R* (Zhang et al. 2019) have been mapped on chromosome 4D. Therefore, the novel QTL detected in the current study provides valuable sources for APR. Accession numbers WLA-106 (Ukraine), WLA-124 (Russia) for *qNV.Yr-1D-1*, WLA-43 (Pakistan) for *qNV.Yr-3D.1* and *qNV.Yr-5D,* and WLA-114 (Azerbaijan) for *QYr.uq-7D.2* represent valuable donor lines that can be used for pre-breeding or future research seeking to fine-map or clone the underlying gene(s).

### Novel APR QTL confer additive effects to reduce disease

We demonstrated that pyramiding of novel alleles associated with APR can significantly reduce YR disease. As a group, APR lines that carry a combination of 2‒3 resistance alleles show stronger levels of resistance compared to lines carrying just one resistance allele. However, allele effects are variable depending on the QTL, the QTL combination, genetic background and environmental conditions. For example, WLA-043 from Pakistan carries three QTL (3A, 3D and 5D) and showed stable resistance across years in comparison with WLA-279 from Kazakhstan which also carries three QTL (1D, 2A and 3A). It has been well described that some resistance genes (such as *Yr18*, *Yr30* and *Yr39*) can increase the effectiveness of other R genes or weak APR genes (Chen et al., 2013, Ellis et al, 2014). While *Yr18* and *Yr49* confer additive effects for YR resistance, increased stem rust severity was detected in Chinese Spring due to the inactivation of *Lr34* gene in the presence of *Sr2* (Ellis et al. 2014). Therefore, it is important to understand gene interactions to incorporate effective disease resistance into wheat varieties.

## Conclusions

This is the first study to document YR resistance in diverse wheat accessions sourced from the VIR. GWAS identified 47 genomic regions including 68 markers that were significantly associated with resistance. Over 90% of the QTL detected in this study aligned with previously reported genes and QTL, which highlights the power of association mapping and accuracy of resistance loci that have been identified in the present study. The molecular markers linked to QTL identified in this study will be valuable for further validation of resistance loci and potentially future marker-assisted selection breeding approaches. Further, insight into the distribution of resistance alleles and their frequencies in relation to geographical origin could aid selection of specific sources of resistance in breeding programmes. The significant effect of pyramiding novel APR alleles identified in this study underscores the potential for breeding wheat varieties with improved YR resistance.

## Supplementary Information

Below is the link to the electronic supplementary material.

Supplementary file1 (XLSX 47 kb)

Supplementary file2 (DOCX 418 kb)

Supplementary file3 (DOCX 168 kb)

Supplementary file4 (XLSX 34 kb)

Supplementary file5 (DOCX 33 kb)

Supplementary file6 (DOCX 133 kb)

Supplementary file7 (XLSX 15 kb)

## Data Availability

The datasets generated during and/or analysed during the current study (i.e. phenotypes, genotypes, and mapping data) are available from the corresponding author on reasonable request.
