# Supplementary material for: Mining the Vavilov wheat diversity panel for new sources of adult plant resistance to stripe rust
Source: Theor Appl Genet. 2022 Feb 3;135(4):1355–73. doi: 10.1007/s00122-022-04037-8 (PMC9033734; doi:10.1007/s00122-022-04037-8)
Supplement: Supplementary file 2 — Supplementary file2 (DOCX 418 kb) [file 122_2022_4037_MOESM2_ESM.docx]

Online resource 2


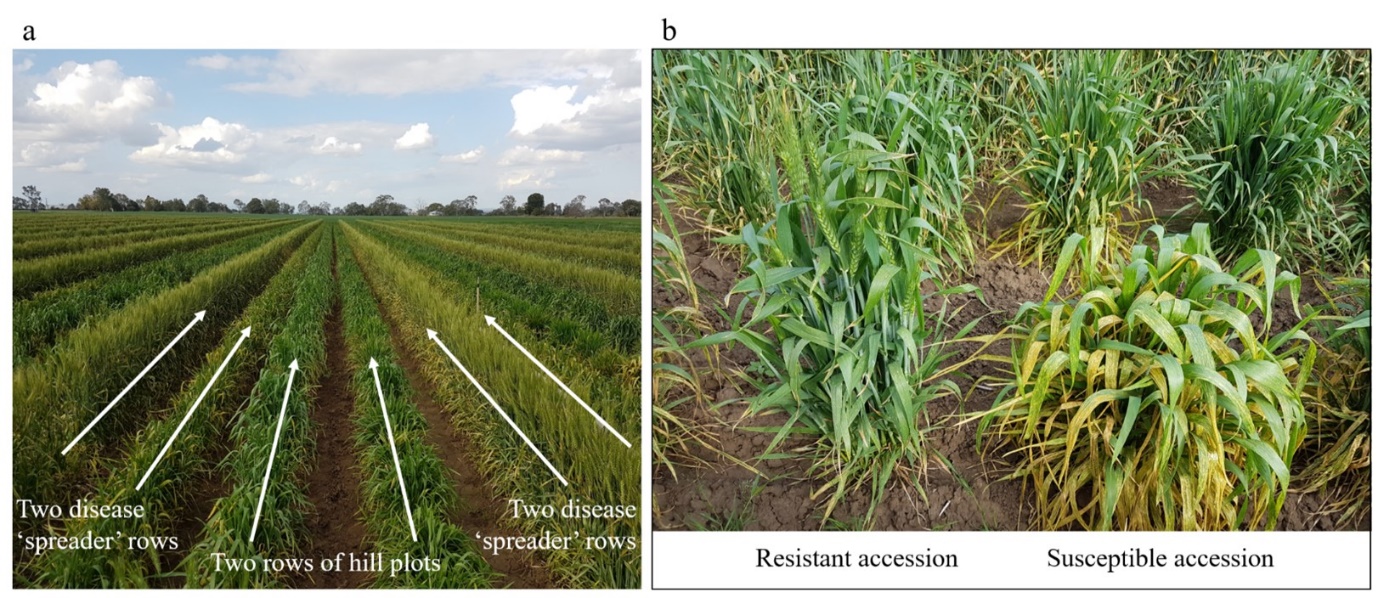


Phenotyping adult wheat plants for resistance to stripe rust in the field. (a) The layout of rust spreader rows and hill plots of test material to maximise infection throughput the screening nursery. (b) Examples of Vavilov wheat accessions showing highly resistant and susceptible responses to stripe rust disease.
