# Supplementary material for: Mining the Vavilov wheat diversity panel for new sources of adult plant resistance to stripe rust
Source: Theor Appl Genet. 2022 Feb 3;135(4):1355–73. doi: 10.1007/s00122-022-04037-8 (PMC9033734; doi:10.1007/s00122-022-04037-8)
Supplement: Supplementary file 3 — Supplementary file3 (DOCX 168 kb) [file 122_2022_4037_MOESM3_ESM.docx]

Online resource 3


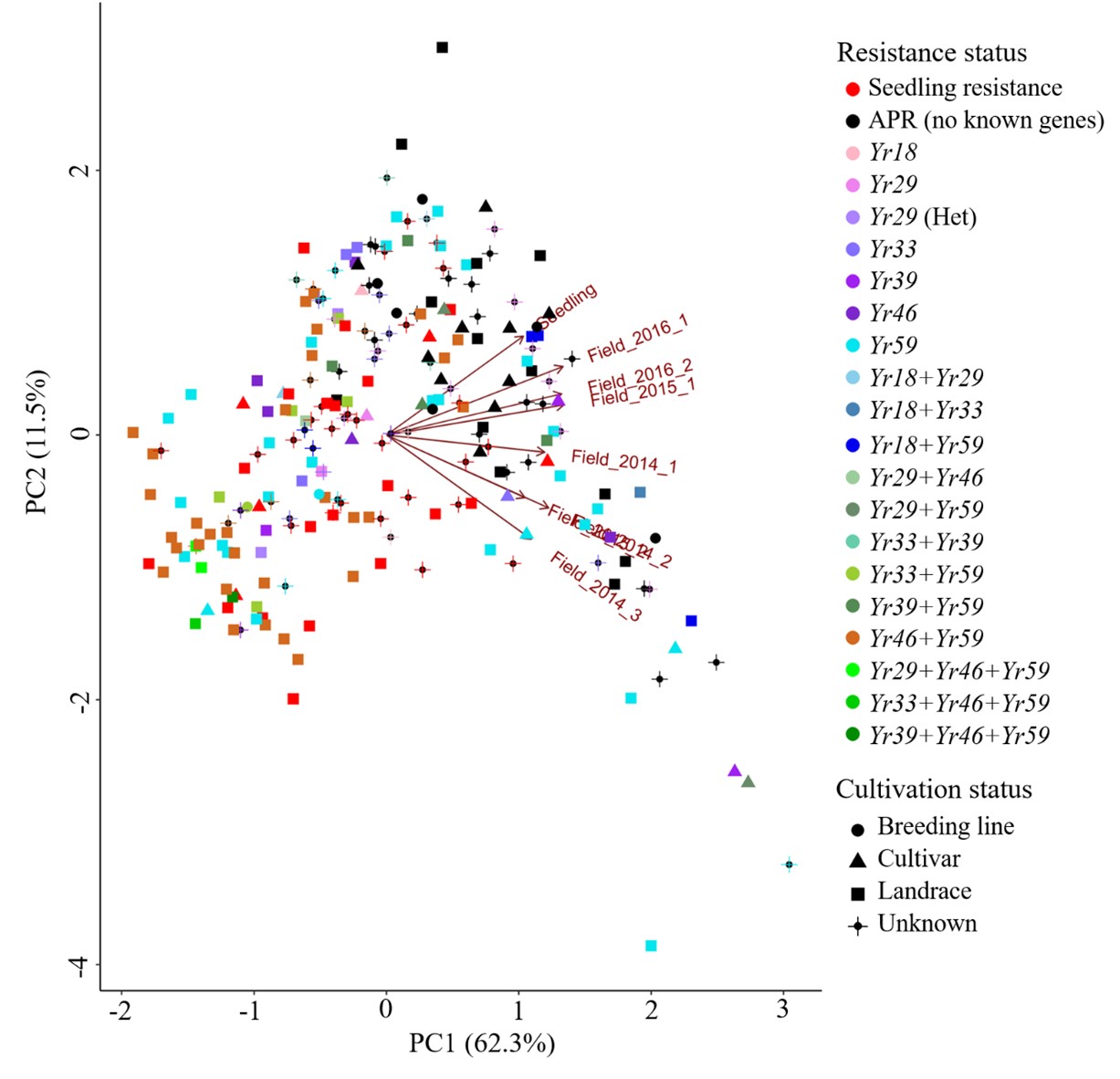


Principal component analysis of wheat accessions using disease response derived from experiments conducted at the seedling stage in the glasshouse and adult growth stages in the field in 2014, 2015 and 2016. The cultivation status of wheat accessions is represented by different shapes coloured according to the presence/absence of known APR genes. Black colour indicates accessions that were susceptible at the seedling stage and lacked known APR genes. Vectors (arrows) illustrate the direction of disease response in each of the eight phenotype datasets using the 0–9 scale (0 = no disease, 9 = very susceptible). The accessions plotted in the direction of the arrows are more susceptible, while accessions plotted in the opposite direction are more resistant. The *x-axis* presents the first principal component (PC1) and the *y-axis* the second (PC2), in total explaining 73.8% of the variance.
