# Supplementary material for: Mining the Vavilov wheat diversity panel for new sources of adult plant resistance to stripe rust
Source: Theor Appl Genet. 2022 Feb 3;135(4):1355–73. doi: 10.1007/s00122-022-04037-8 (PMC9033734; doi:10.1007/s00122-022-04037-8)
Supplement: Supplementary file 5 — Supplementary file5 (DOCX 33 kb) [file 122_2022_4037_MOESM5_ESM.docx]

**Online Resource 5**

Alignment of putative QTL to previously reported *Yr* genes and QTL.

Chromosome 1 B

Two QTL on chromosome 1B, *qNV.Yr-1B-1* and *qNV.Yr-1B-2* were detected in the field (Field_2016_2) and mapped to the distal end of chromosome 1B, at positions closed to the loci (IWA3892, IWA2077) identified by Maccaferri et al. (2015). It supports the hypothesis of presence of more than one *Pst* resistance gene in this region (Maccaferri et al. 2015, Rosewarne et al. 2013). The *QTL qNV.Yr-1B-2* identified by DArT marker 1119286 at position 269.3 cM corresponds better with the HTAP resistant locus *Yr29/Lr46* (Maccaferri et al. 2015; Lan et al. 2015).

Chromosome 1 D

The QTL *qNV.Yr-1D-1* was identified by DArT marker 1862252 at position 29.7 cM on chromosome 1D which was 10 cM away from the QTL *QYr.ucw‐1D* (Maccaferri et al. 2015) thus was considered novel. QTL, *QYr.uq-1D.2* detected in the field (Field_2016) at position of 47.7 cM and collocated closed proximity to the QTL *QYr.ucw‐1D* (Maccaferri et al. 2015).

Chromosome 2 A

Six QTL were detected in chromosome 2A representing five reported *Pst* resistant loci and one novel locus. The first QTL *qNV.Yr-2A.1* (9.93 cM) was collocated with QYr.ucw‐2A.2 = IWA422 (Maccaferri et al. 2015). The second QTL *qNV.Yr-2A.2* mapped closed to the gene *Yr56*. However, separation of these QTL is complicated because this region represents the ancestral R gene cluster (Maccaferri et al. 2015). For example, more than ten QTL have been identified in this region (Vazquez et al. 2015; Agenbag et al. 2012; Naruoka et al. 2015; Basnet et al. 2014b). All these QTL and *Yr56* gene located within the distance 17% of chromosome 2A (Maccaferri et al. 2015). The other two QTL *qNV.Yr-2A.3* (46.8 cM) and *qNV.Yr-2A.4* (60.5 cM) detected in the field (Field_2015) collocated with the locus IWA 2059 (Naruoka et al. 2015) and IWA2526 (Bulli et al. 2016a) respectively. However, in their paper they could not establish definite relationship between these QTL and any previously reported gene or QTL. The QTL *qNV.Yr-2A.5* (68.6 cM) detected in the field (Field_2014_1 and Field_2015_ 2) and did not collocate with any previously reported gene or QTL. This QTL potentially novel but, need to be confirmed with allelism test. *qNV.Yr-2A.6* detected in the field 2014 and collocated with the QTL *QYr.inra-2AL* (Dedryver et al. 2009).

Chromosome 2 B

The position of *qNV.Yr-2B.1* falls within the confidence interval of *QYr.tsw-2B.1*(Godoy et al. 2017) on the integrated map which is proximity to the gene *Yr5*. The *Yr5* gene is one of the major genes with rare virulence worldwide except the virulence for some pathotypes in Australia and India (Zegeye et al. 2014). However, in Australia, *Yr5* gene is avirulence for *Pst* pathotype 134 E16 A+ Yr17 +Yr27 (Cereal rust report 2018). Therefore, the QTL, *qNV.Yr-2B.1* is likely to be the gene *Yr5*. The other all stage resistance *Yr* genes, including *Yr44*, *Yr53* and *Yr43* have been reported in the long arm of chromosome 2B (Xu et al. 2012a; McGranna et al. 2014). The QTL*, qNV.Yr-2B.2* identified at the seedling and adult plant stages (2015, 2016) with seven significant markers and mapped within the confidence interval of previously mapped *Yr43* gene (Maccaferri et al. 2015a, Xu et al. 2012a). It is therefore, *qNV.Yr-2B.2* is likely *Yr43* gene. The QTL *qNV.Yr-2B.3* (107.0 cM) was contributed strong marker effect for *Pst* resistance among all other QTL at chromosome 2B. It was detected in two field environments (Field_2_2014, Field_2_2015) and overlapped with the interval of *QYr.ifa-2BL* at marker wPt-733641(Buerstmayr et al. 2014) and wPt-3378 (Crossa et al. 2007b) which has been identified as important genomic region associated with both stripe rust and leaf rust resistance. However, no designated *Yr* gene has been reported so far distal end of chromosome 2B.

Chromosome 2D

Two QTL *qNV.Yr-2D.1*, *qNV.Yr-2D.2* were detected on chromosome 2D by DArT markers 2246033, 2338436 (70.8 cM) and 2243695 (136.4 cM) respectively. Both QTL were located on *QYR2D.2* region (Rosewarne et al. 2013) which is known as large genomic region with several QTL from different studies (Ren et al., 2012, Powell et al. 2013; Naruoka et al. 2015; Mallard et al. 2005). However, differentiate of these QTL is difficult due to lack of genetic map to compare relative distances. Further work would be required to understand this valuable genomic region.

Chromosome 3A

Five QTL detected on chromosome 3A. QTL *qNV.Yr-3A.1* (44.75 cM) detected at seedling stage, and it mapped closed to the marker IWA8630 (Zegeye et al. 2014). *QTL qNV.Yr-3A.2* (49.2 cM) detected in Field_1_2015 and co-located with the confidence interval of SNPs IWA7440 and IWA7441(Pasam et al. 2017). *QTL qNV.Yr-3A.3* mapped closed to the HTAP QTL *QYrdr.wgp-3AL* (Hou et al., 2015). In this region of chromosome 3A limited number of Yr genes or QTL detected so far. Therefore, it is unclear whether these QTL represent same QTL, allelic form or different QTL. *qNV.Yr-3A.4* maps in the same chromosomal region of the *QYr.cim‐3A_Avocet* (Rosewarne et al. 2012). However, *qNV.Yr-3A.5* (142.1cM) did not align with any previously detected gene or QTL, thus was considered novel.

Chromosome 3B

QTL, *qNV.Yr-3B* (5.0 cM) detected in field 2016 (Field_2016_1, Field_2016_2) and seedling stage with DArT markers 2307351 and 1239212. This QTL mapped closed proximity to the genomic region of *QYr.cim‐3BS.2_Frankolin* (Lan et al, 2014), *QYr.tam-3B* (Basanet et al. 2013). The *Yr57* gene and slow rusting resistance gene *Yr30* which is known as pleotropic to stem rust resistance gene *Sr2* also present in the same chromosome region. It is concluded that this region may be an ancestral R gene cluster (Maccaferri et al. 2015) and further studies required to confirm the exact relationship between each Yr gene and QTL.

Chromosome 3D

*qNV.Yr-3D.1* (59.5 cM) detected in the field (Field_2014_1) and did not collocate with any previously identified gene or QTL, Thus, was considered novel. *qNV.Yr-3D.2* (123.8cM) mapped closed to the Avocet derived minor *QTL QYr.tam-3D.1* (Basenet et al. 2013).

Chromosome 4A

*qNV.Yr-4A.1* (96.1 cM) detected in the field (Field_2014_2) was closely linked with locus identified by IWA3774 (Bulli et al. 2016a) and IWA8475 (Zegeye et al. 2014). The locus IWA 3774 identified as important novel QTL for future breeding as it was only present in high frequencies in landraces (Bulli et al, 2016) compared to other lines. The QTL*, qNV.Yr-4A.2* detected in close to the QTL *QYrst.orr-4AL* reported by (Vazquez et al. 2012; Chen et al. 2012).

Chromosome 4D

The QTL *qNV.Yr-4D.1* (43.4 cM) detected in the field (Field_2014_2, Field_2016_1, Field_2016_2) and collocated with *Yr46* gene (Herrera-Foessel et al. 2011). *qNV.Yr-4D.2* identified in field_2014_1 and mapped closed to the QTL *QYr.caas-4DL* (Ren et al. 2012).

Chromosome 5A

The QTL *qNV.Yr-5A* detected in seedling stage seem to be the same QTL reported by (Manickavelu et al. 2016).

Chromosome 5B

Four QTL have been detected in chromosome 5B. QTL *qNV.Yr-5B.1* (12.4 cM) detected in the field (Field_2015_1) collocated with the confidence interval of markers wPt-8604 and wPt-9666 which represent the *Yr47* and *Lr52* gene (Bansal et al 2013). *qNV.Yr-5B.2* detected in the field (Field_2016_2) by marker 1125706, collocated with the QTL *QYr.uga‐5B_AGS2000* (Hao et al. 2011). *qNV.Yr-5B.3* (27.8-29.32 cM) identified by two markers in the field (Field_2015_2) and mapped closed proximity to *QYr.cim‐5BL_Chapio* (Yang et al. 2013) detected in the Chinese environment. The small effect of HTAP resistance locus (*QYrPI192252.wgp-5BS*) detected by (Lu et al. 2014) also collocated within the confidence interval of *qNV.Yr-5B.3*. Further studies would be required to identify whether *qNV.Yr-5B.3* represents only one locus or closely linked loci. *qNV.Yr-5B.4* identified by marker 1107669 seems to be same locus of *QYr.caas-5BL.1* and *QYrns.orz-5BL* reported by Lu et al. 2009 and Vazquez et al. 2015 respectively.

Chromosome 5D

*qNV.Yr-5D* (58.6 cM) on chromosome 5D detected in the field (Field_2014_1,) by marker 991465 did not collocate with any previously identified gene or QTL, Thus, was considered novel.

Chromosome 6A

*qNV.Yr-6A* detected in the field (Field_2016_2) by marker 3022417 maps to the proximal region of Qyr.wsu-6A (Bulli et al. 2016) and *Qyrpl.orr-6AL* (Vazquez et al. 2012) on chromosome 6AL.

Chromosome 6B

In chromosome 6B five QTL have been detected. The confidence interval of markers in *qNV.Yr-6B.1*(2.5-4.5cM) and *qNV.Yr-6B.2* (10.89 cM) had overlapped each other, thus the relationship between them could not established. However, both QTL collocated with *Yr35* gene. Another two QTL were also detected in the same region by marker wPt-7745 (Prins et al. 2011; Bansal et al. 2013). It suggests that there can be more than one resistant loci in this region. *qNV.Yr-6B.3* identified close proximity to locus IWA3473 detected by Passam et al. 2017. It was also within the confidence interval of *QYrst.wgp-6BS.1*(Santra et al. 2008), *QYr.sun-6B* (Bariana et al. 2010) and *Yr36* gene. However, HTAP resistant locus *QYrst.wgp-6BS.1* derived from Stephens and *Yr36* are different from each other (Santra et al. 2008). It suggests that more than one HTAP resistant loci present in this region. *qNV.Yr-6B.4* identified by marker 1058394 collocated with novel APR QTL, *Qrfi.wak-6B* (Klarquist et al. 2016) identified by US Pacific Northwest soft white winter wheat variety “Finch”. *QYr.uq-6B.5* (79.7 -82.95 cM) identified in the field (Field_2014_3, Field_2015_1, Field_2016_2) seems to be a same QTL, *QYr.cim‐6BL_Pastor*(wPt-5176) reported by Roswarne et al. 2012 and *QYr.tam-6B* (wPt-4164) reported by Basenet et al. 2013 from Quaiu.

Chromosome 7A

*qNV.Yr-7A.1*(96.1 cM) identified in the field (Field_2015_2) mapped closed to the *Yrxy1* gene (Zhou et al. 2011) identified from Chinese cultivar Xiaoyan 54. *qNV.Yr-7A.2* (149.5cM) located in the same confidence interval of QTL, *QYrst.orr-7AS-Stephans* (Vazquez et al., 2012), *QYr.sgi‐7A_Kariega* (Prins et al. 2011), *QYrTtd-7AL.2* (Liu et al. 2017) and Avocet derived *QTL,* *QYr.cim-7AL* from Avocet X Paster population (Roswarne et al. 2012). But QTL with marker IWA 501 (*QYrTtd-7AL.2*) and markers wPt-2260 and wPt-2501 (*QYr.cim-7AL*) expressed major and minor effect on stripe rust resistance (Liu et al. 2017). It suggests that there could be more than one resistant loci in this region.

Chromosome 7B

Four different genomic regions for stripe rust resistance have been identified on chromosome 7B. *qNV.Yr-7B.1* (21 cM) on chromosome 7B detected in the field (Field_2014_2) by marker 1126816 did not collocate with any previously identified gene or QTL, Thus, was considered novel. *qNV.Yr-7B.2* (38.7cM) identified by marker 1059624 and 1259637 mapped closed to the locus identified by gwm935.3 (Suanaga et al. 2003). *qNV.Yr-7B.3* identified by markers 1298605, 3026338 and 1159261 and correspond with the position of marker IWA1971 (Pasam et al. 2017). *qNV.Yr-7B.4* (118.5 cM) was overlap with the genomic region of *Yr52* (Ren et al. 2012), *Yr59* (Zhou et al. 2014) and *YrC591* (Wheat catalogue) and QTL (Manickavelu et al. 2016; Leu et al. 2017). Therefore, *qNV.Yr-7B.4* could be belonging to any of the gene reported. Allelism testing would be required to verify gene.

Chromosome 7D

Two QTL were identified on chromosome 7D in 2 different environments. The QTL *qNV.Yr-7D.1* detected by the marker 1105401 in the field (Field_2_2015) mapped closed to the novel all stage stripe rust resistance gene *YrYL* derived from Chinese landrace “Yilongtuomai” (Wu et al. 2016). The QTL *qNV.Yr-7D.2* detected in the field (Field_1_2016) significantly associated with YR resistance (p=3.3) and did not collocate with any previously reported gene or QTL, thus was considered novel.

**References**

Agenbag GM, Pretorius ZA, Boyd LA, Bender CM, Prins R (2012) Identification of adult plant resistance to stripe rust in the wheat cultivar Cappelle-Desprez. Theor Appl Genet 125**:**109-20. https://doi.org/ 10.1007/s00122-012-1819-5

Bansal UK, Arief VN, Delacy IH, Bariana HS (2013) Exploring wheat landraces for rust resistance using a single marker scan. Euphytica, 194: 219-233. https://doi.org/10.1007/s10681-013-0940-0

Basnet BR, Ibrahim AM, Chen X, Singh RP, Mason ER, Bowden RL, Liu S, Hays DB, Devkota RN, Subramanian NK (2014b) Molecular mapping of stripe rust resistance in hard red winter wheat TAM 111 adapted to the US High Plains. Crop Sci 54:1361-1373. https://doi.org/10.2135/cropsci2013.09.0625

Buerstmayr M, Matiasch L, Mascher F, Vida G, Ittu M, Robert O, Holdgate S, Flath K, Neumayer A, Buerstmayr H (2014) Mapping of quantitative adult plant field resistance to leaf rust and stripe rust in two European winter wheat populations reveals co-location of three QTL conferring resistance to both rust pathogens. Theor Appl Genet 127**:**2011-28. https://doi.org/10.1007/s00122-014-2357-0

Bulli, P., Zhang, J., Chao, S., Chen, X. & Pumphrey, M (2016a) Genetic architecture of resistance to stripe rust in a global winter wheat germplasm collection. G3 6:2237-2253. https://doi.org/10.1534/g3.116.028407

Chen J, Chu C, Souza EJ, Guttieri MJ, Chen X, Xu S, Hole D, Zemetra R (2012) Genome-wide identification of QTL conferring high-temperature adult-plant (HTAP) resistance to stripe rust (*Puccinia striiformis* f. sp*. tritici*) in wheat. Mol Breed*,* 29**:**791-800. https://doi.org/10.1007/s11032-011-9590-x

Crossa J, Burgueno J, Dreisigacker S, Vargas M, Herrera-Foessel SA, Lillemo M, Singh RP, Trethowan R, Warburton M, Franco J (2007) Association analysis of historical bread wheat germplasm using additive genetic covariance of relatives and population structure. J Genet 177:1889-1913. https://doi.org/10.1534/genetics.107.078659

Dedryver F, Paillard S, Mallard S, Robert O, Trottet M, Negre S, Verplancke G, Jahier J (2009) Characterization of genetic components involved in durable resistance to stripe rust in the bread wheat 'Renan'. Phytopathology 99**:**968-73. https://doi.org/10.1094/PHYTO-99-8-0968

Dolores Vazquez M, James Peterson C, Riera-Lizarazu O, Chen X, Heesacker A, Ammar K, Crossa J, Mundt C (2012) Genetic analysis of adult plant quantitative resistance to stripe rust in wheat cultivar 'Stephens' in multi-environment trials. Theor Appl Genet 124:1-11. https://doi.org/10.1007/s00122-011-1681-x

Godoy JG, Rynearson S, Chen X, Pumphrey M (2017) Genome-wide association mapping of loci for resistance to stripe rust in North American elite spring wheat germplasm. Phytopathology 108**:**234-245. https://doi.org/10.1094/PHYTO-06-17-0195-R

Herrera-Foessel S A, Lagudah ES, Huerta-Espino J, Hayden MJ, Bariana HS, Singh D, Singh RP (2011) New slow-rusting leaf rust and stripe rust resistance genes *Lr67* and *Yr46* in wheat are pleiotropic or closely linked. Theor and Appl Genet 122:239-249. https://doi.org/10.1007/s00122-010-1439-x

Hou L, Chen X, Wang M, See DR, Chao S, Bulli P, Jing J (2015) Mapping a large number of qtl for durable resistance to stripe rust in winter wheat Druchamp using SSR and SNP markers. *PLoS One,* 10**:**e0126794 https://doi.org/10.1371/journal.pone.0126794

Klarquist FE, Chen MX, Carter HA (2016) Novel QTL for stripe rust resistance on chromosomes 4A and 6B in soft white winter wheat cultivars. Agronomy 6. https://doi.org/10.3390/agronomy6010004

Lan C, Zhang Y, Herrera-Foessel SA, Basnet BR, Huerta-Espino J, Lagudah ES, Singh RP (2015) Identification and characterization of pleiotropic and co-located resistance loci to leaf rust and stripe rust in bread wheat cultivar Sujata. Theor Appl Genet 128:549-561. https://doi.org/10.1007/s00122-015-2454-8

Liu W, Maccaferri M, Bulli P, Rynearson S, Tuberosa R, Chen X, Pumphrey M (2017) Genome-wide association mapping for seedling and field resistance to *Puccinia* *striiformis* f. sp. *tritici* in elite durum wheat. Theor Appl Genet 130:649-667. https://doi.org/10.1007/s00122-016-2841-9

Lu Y, Lan C, Liang S, Zhou X, Liu D, Zhou G, Lu Q, Jing J, Wang M, Xia X, He Z (2009) QTL mapping for adult-plant resistance to stripe rust in Italian common wheat cultivars Libellula and Strampelli. Theor Appl Genet 119**:**1349-59. https://doi.org/10.1007/s00122-009-1139-6

Lu Y, Wang M, Chen X, See D, Chao S, Jing J (2014) Mapping of *Yr62* and a small-effect QTL for high-temperature adult-plant resistance to stripe rust in spring wheat PI 192252. Theor Appl Genet 127:1449-59. https://doi.org/10.1007/s00122-014-2312-0

Maccaferri M, Ricci A, Salvi S, Milner SG, Noli E, Martelli PL, Casadio R, Akhunov E, Scalabrin S, Vendramin V, Ammar K, Blanco A, Desiderio F, Distelfeld A, Dubcovsky J, Fahima T, Faris J, Korol A, Massi A, Mastrangelo AM, Morgante M, Pozniak C, N'Diaye A, Xu S, Tuberosa, R (2015a) A high-density, SNP-based consensus map of tetraploid wheat as a bridge to integrate durum and bread wheat genomics and breeding. Plant Biotechnol J 13:648-663. https://doi.org/10.1111/pbi.12288

Maccaferri M, Zhang J, Bulli P, Abate Z, Chao S, Cantu D, Bossolini E, Chen X, Pumphrey M, Dubcovsky J (2015b) A genome-wide association study of resistance to stripe rust (*Puccinia* *striiformis* f. sp. *tritici*) in a worldwide collection of hexaploid spring wheat (*Triticum* *aestivum* L.). G3 (Bethesda), 5:449-65. https://doi.org/10.1534/g3.114.014563

Mcgrann G, Smith P, Burt C, Mateos G, Chama T, Maccormack R, Wessels E, Agenbag G (2014) Genomic and genetic analysis of the wheat race-specific yellow rust resistance gene *Yr5*. J Plant Sci Mol Bree 3:2. https://doi.org/10.7243/2050-2389-3-2

Mallard S, Gaudet D, Aldeia A, Abelard C, Besnard AL, Sourdille P, Dedryver F (2005) Genetic analysis of durable resistance to yellow rust in bread wheat. Theor Appl Genet 110:1401-9. https://doi.org/10.1007/s00122-005-1954-3

Manickavelu A, Joukhadar R, Jighly A, Lan C, Huerta-Espino J, Stanikzai A S, Kilian A, Singh RP, Ban T (2016) Genome wide association mapping of stripe rust resistance in Afghan wheat landraces. Plant Sci 252:222-229. https://doi.org/10.1016/j.plantsci.2016.07.018

Naruoka Y, Garland-Campbell KA, Carter AH (2015) Genome-wide association mapping for stripe rust (*Puccinia* *striiformis* F. sp. *tritici*) in US Pacific Northwest winter wheat (*Triticum* *aestivum* L.). Theor Appl Genet 128:1083-101. https://doi.org/10.1007/s00122-015-2492-2

Pasam RK, Bansal U, Daetwyler HD, Forrest KL, Wong D, Petkowski J, Willey N, Randhawa M, Chhetri M, Miah H, Tibbits J, Bariana H, Hayden MJ (2017) Detection and validation of genomic regions associated with resistance to rust diseases in a worldwide hexaploid wheat landrace collection using BayesR and mixed linear model approaches. Theor Appl Genet 130:777–793. https://doi.org/10.1007/s00122-016-2851-7

Powell NM, Lewis CM, Berry ST, Maccormack R, Boyd LA (2013) Stripe rust resistance genes in the UK winter wheat cultivar Claire. Theor Appl Gene 126**:**1599-612. https://doi.org/10.1007/s00122-013-2077-x

Prins R, Pretorius Z, Bender C, Martin A (2011) QTL mapping of stripe, leaf and stem rust resistance genes in a Kariega × Avocet S doubled haploid wheat population. Mol Breed 27**:**259-270. https://doi.org/10.1007/s11032-010-9428-y

Ren Y, Li Z, He Z, Wu L, Bai B, Lan C, Wang C, Zhou G, Zhu H, Xia X (2012) QTL mapping of adult-plant resistances to stripe rust and leaf rust in Chinese wheat cultivar Bainong 64. Theor Appl Genet 125:1253-62. https://doi.org/10.1007/s00122-012-1910-y

Rosewarne GM, Herrera-Foessel SA, Singh RP, Huerta-Espino J, Lan CX, He ZH (2013) Quantitative trait loci of stripe rust resistance in wheat. Theor Appl Genet 126:2427-49. https://doi.org/10.1007/s00122-013-2159-9

Rosewarne GM, Singh RP, Huerta-Espino J, Herrera-Foessel SA, Forrest KL, Hayden MJ, Rebetzke GJ (2012) Analysis of leaf and stripe rust severities reveals pathotype changes and multiple minor QTLs associated with resistance in an Avocet x Pastor wheat population. Theor Appl Genet 124:1283-94. https://doi.org/10.1007/s00122-012-1786-x

Santra DK, Chen XM, Santra M, Campbell KG, Kidwell KK (2008) Identification and mapping QTL for high-temperature adult-plant resistance to stripe rust in winter wheat (*Triticum aestivum* L.) cultivar 'Stephens'. Theor Appl Genet 117:793-802. https://doi.org/10.1007/s00122-008-0820-5

Suenaga K, Singh RP, Huerta-Espino J, William HM (2003) Microsatellite markers for genes lr34/yr18 and other quantitative trait Loci for leaf rust and stripe rust resistance in bread wheat. Phytopathology 93:881-90. https://doi.org/10.1094/PHYTO.2003.93.7.881

Vazquez MD, Zemetra R, Peterson CJ, Chen XM, Heesacker A, Mundt CC (2015) Multi-location wheat stripe rust QTL analysis: genetic background and epistatic interactions. Theor Appl Genet 128:1307-18. https://doi.org/10.1007/s00122-015-2507-z

Wu XL, Wang JW, Cheng YK, Ye XL, Li W, Pu ZE, Jiang QT, WeI YM, Deng M, Zheng YL, Chen GY (2016) Inheritance and molecular mapping of an all-stage stripe rust resistance gene derived from the Chinese common wheat landrace "Yilongtuomai". J Hered 107:463-70. https://doi.org/10.1093/jhered/esw032

Xu LS, Wang MN, Cheng P, Kang Z, Hulbert S, Chen X (2013) Molecular mapping of *Yr53*, a new gene for stripe rust resistance in durum wheat accession PI 480148 and its transfer to common wheat. Theor and Appl Genet 126:523-533. https://doi.org/10.1007/s00122-012-1998-0

Yang EN, Rosewarne GM, Herrera-Foessel SA, Huerta-Espino J, Tang ZX, Sun CF, Ren ZL, Singh RP (2013a) QTL analysis of the spring wheat "Chapio" identifies stable stripe rust resistance despite inter-continental genotype x environment interactions. Theor Appl Genet 126:1721-32. https://doi.org/10.1007/s00122-013-2087-8

Zegeye H, Rasheed A, Makdis F, Badebo A, Ogbonnaya FC (2014) Genome-wide association mapping for seedling and adult plant resistance to stripe rust in synthetic hexaploid wheat. PLoS One 9. https://doi.org/10.1371/journal.pone.0105593

Zhou XL, Wang MN, Chen XM, Lu Y, Kang ZS, Jing JX (2014) Identification of *Yr59* conferring high-temperature adult-plant resistance to stripe rust in wheat germplasm PI 178759. Theor Appl Genet 127: 935-45. https://doi.org/10.1007/s00122-014-2269-z
