# Supplementary material for: Mining the Vavilov wheat diversity panel for new sources of adult plant resistance to stripe rust
Source: Theor Appl Genet. 2022 Feb 3;135(4):1355–73. doi: 10.1007/s00122-022-04037-8 (PMC9033734; doi:10.1007/s00122-022-04037-8)
Supplement: Supplementary file 6 — Supplementary file6 (DOCX 133 kb) [file 122_2022_4037_MOESM6_ESM.docx]

Online Resource 6

Genomic regions associated with stripe rust resistance in the Vavilov diversity panel projected on the integrated consensus map developed by Macafferri et al, (201).
